# Evolution and regulation of nitrogen flux through compartmentalized metabolic networks in a marine diatom

**DOI:** 10.1038/s41467-019-12407-y

**Published:** 2019-10-07

**Authors:** Sarah R. Smith, Chris L. Dupont, James K. McCarthy, Jared T. Broddrick, Miroslav Oborník, Aleš Horák, Zoltán Füssy, Jaromír Cihlář, Sabrina Kleessen, Hong Zheng, John P. McCrow, Kim K. Hixson, Wagner L. Araújo, Adriano Nunes-Nesi, Alisdair Fernie, Zoran Nikoloski, Bernhard O. Palsson, Andrew E. Allen

**Affiliations:** 1 Microbial and Environmental Genomics, J. Craig Venter Institute, La Jolla, CA 92037 USA; 2 Division of Biological Sciences, University of California, San Diego, La Jolla, CA 92093 USA; 3 Department of Bioengineering, University of California, San Diego, La Jolla, CA 92093 USA; 4 Institute of Parasitology, Biology Centre Czech Academy of Sciences, Branišovská 31, 370 05 České Budějovice, Czech Republic; 5 Faculty of Science, University of South Bohemia, Branišovská 31, 370 05 České Budějovice, Czech Republic; 6 Targenomix, GmbH, Wissenschaftspark Potsdam-Golm, 14476 Potsdam, Germany; 7 Environmental Molecular Sciences Laboratory, Pacific Northwest National Laboratory, Richland, WA 99352 USA; 8 Departamento de Biologia Vegetal, Universidade Federal de Viçosa, Viçosa, Minas Gerais 36570-900 Brazil; 9 Max-Planck Partner Group at the Departamento de Biologia Vegetal, Universidade Federal de Viçosa, Viçosa, Minas Gerais 36570-900 Brazil; 10 Max Planck Institut of Molecular Plant Physiology, Am Mühlenberg 1, 14476 Potsdam, Germany; 11 Institute of Biochemistry and Biology, University of Potsdam, Karl-Liebknecht-Str. 24-25, 14476 Potsdam, Germany; 12 Scripps Institution of Oceanography, Integrative Oceanography Division, University of California, San Diego, La Jolla, CA 92093 USA

**Keywords:** Biochemistry, Computational biology and bioinformatics, Evolution, Microbiology, Molecular biology

## Abstract

Diatoms outcompete other phytoplankton for nitrate, yet little is known about the mechanisms underpinning this ability. Genomes and genome-enabled studies have shown that diatoms possess unique features of nitrogen metabolism however, the implications for nutrient utilization and growth are poorly understood. Using a combination of transcriptomics, proteomics, metabolomics, fluxomics, and flux balance analysis to examine short-term shifts in nitrogen utilization in the model pennate diatom in *Phaeodactylum tricornutum*, we obtained a systems-level understanding of assimilation and intracellular distribution of nitrogen. Chloroplasts and mitochondria are energetically integrated at the critical intersection of carbon and nitrogen metabolism in diatoms. Pathways involved in this integration are organelle-localized GS-GOGAT cycles, aspartate and alanine systems for amino moiety exchange, and a split-organelle arginine biosynthesis pathway that clarifies the role of the diatom urea cycle. This unique configuration allows diatoms to efficiently adjust to changing nitrogen status, conferring an ecological advantage over other phytoplankton taxa.

## Abstract (web summary)

Here, using the diatom *Phaeodactylum tricornutum* as a model organism, the authors combine functional genomics, phylogenetics, and metabolic modeling to describe how diatoms might have functionally integrated nitrogen metabolism during evolution and how metabolic flux is regulated across cellular compartments

## Introduction

Diatoms are photosynthetic microbes that contribute substantially to global primary production in aquatic habitats^1^. They are widely distributed in sunlit ocean ecosystems and are particularly successful in high or dynamic nutrient environments such as upwelling and high nutrient low chlorophyll (HNLC) systems. The high growth rate, rapid nutrient uptake rates, and capacity for nitrate uptake beyond immediate metabolic demand, i.e., luxury uptake, are defining functional traits of diatoms relative to other microalgae that are believed to underlie their ecological success^2^. Despite decades of studies aimed at developing a mechanistic understanding of how diatoms achieve this success relative to other phytoplankton taxa, little is known about the genetic components involved in sensing, regulation, and assimilation of different forms and concentrations of nitrogen.

Regulation of nitrogen metabolism is complex and is only beginning to be unraveled in model organisms, like filamentous fungi, green algae, and terrestrial plants^3–5^. While certain elements of nitrogen metabolism and metabolic regulation that have been described are shared within closely related organisms, canonical regulators are not shared across diverse eukaryotes. Diatoms are evolutionarily distant from fungi, green algae, and plants. They originated via secondary endosymbiosis and modern diatom genomes represent a combination of traits from the ancestral heterotrophic stramenopile host, the endosymbiont of the red-algal lineage, and those acquired from bacteria.

Analyses of diatom genomes have revealed that their overall network of nitrogen metabolism is a combination of plant-like, animal-like, and bacterial traits^6,7^. The nitrate assimilation pathway is similar to that from plants^8^, but it was surprising to find that diatoms possess a metazoan-like urea cycle^6^. In animals, the urea cycle functions to excrete excess nitrogen, so its role in phytoplankton was initially unclear. Further investigation in the model diatom *Phaeodactylum tricornutum* provided clarity about the role of the urea cycle in redistribution of central nitrogen-containing metabolites during fluctuating nitrogen availability^9^. Yet, there are still many open questions about the magnitude of and timing of flux through the cycle and how it is integrated within the rest of metabolism under shifting cellular states.

There has been growing interest in the nitrogen metabolism of diatoms because of their industrial potential as a feedstock for biofuels, feeds, or other high-value compounds^10^. Nitrogen limitation induces the accumulation of neutral lipids in diatoms and other algae, and several studies have investigated the impact of nitrogen limitation on gene expression and physiology to better understand and optimize this phenomenon^11–14^. Despite this progress, there are critical gaps in our knowledge of the organization, origins, regulation, and function of nitrogen acquisition and metabolism in diatoms that obscures interpretation of the results of these kinds of studies and hinders strategies to improve desired outcomes.

The availability of tools in the post-genomic era, such as genome-scale metabolic models, provides new opportunities to address some of the key knowledge gaps in diatom nitrogen metabolism^15^. In this study, we combined genome-enabled experimental approaches with genome-scale metabolic modeling to make predictions of metabolite flux leading to an improved understanding of the physiological consequences of nitrogen metabolic network connectivity in diatoms. We sought to establish an improved knowledge of the chimeric nature and pathway organization that contribute directly to the functional traits of marine diatoms that define their ecological niche and to inform efforts aimed at development of biofuels or other bioproducts from diatoms.

## Results

### Identification of highly nitrate-sensitive (HNS) genes

Nitrogen status strongly impacts gene expression, yet little is known about how diatoms respond to specific nitrogen sources at the molecular level. Traditionally, studies on the impacts of variable nitrogen source on gene expression have been valuable in identifying molecular components of nitrate and nitrogen sensing and assimilation^5^. Therefore, we took a similar approach in *P. tricornutum* and evaluated gene expression in an experiment designed to elicit shifts in nitrogen source and status on short timescales (N_short,_ Supplementary Data 1). Cultures were grown to mid-exponential phase on ammonium and sampled, transferred to medium with no nitrogen source for 2 h and sampled (N-pretreatment), and spiked with nitrate (150 µM) for 90 min and sampled again. Following pretreatment, cells were transferred into four different nitrogen conditions: no nitrogen or 300 µM of either ammonium, nitrite, or nitrate, and sampled after 15 min, 45 min, and 18 h. This design, based on previous experiments, induces the expression of nitrate-responsive genes during the pretreatment and ensures that external N sources will be depleted by 18 h^16,17^.

**Fig. 1:**
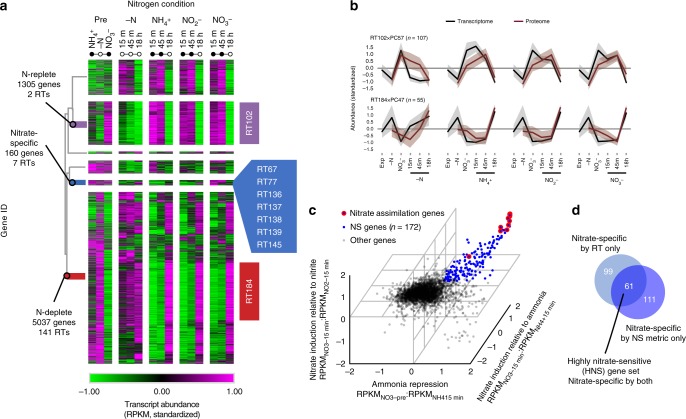
Identification of highly nitrate-sensitive (HNS) gene set. **a** Transcriptome from the N_short_ experiment. Heatmap shows average standardized transcript abundance for all genes in each response type (RT, *n* = 200, RT3 omitted). Dendrogram shows hierarchical clustering of RTs (one-minus Pearson correlation, average linkage). Circles indicate time points when nitrogen in the media is available (closed) or unavailable (open). **b** Standardized average transcriptome and proteome expression of the subsets of genes found in RT102xPC57 and RT184xPC47. Shading indicates standard deviation of standardized RPKM. **c** Scatter plot showing specificity of transcript-level response to nitrate for all genes, in three dimensions (“Methods”). Genes considered to be nitrate specific (NS) in all three dimensions are shown in blue, with known nitrate assimilation genes identified (red circle). **d** The highly nitrate-sensitive (HNS) gene set identified as genes that are in nitrate-sensitive RTs and considered NS. Source data are provided as a Source Data file

The N_short_ transcriptome was analyzed by hierarchically clustering expression patterns and binning the resulting dendrogram to yield 201 distinct clusters or response types (RT1–RT201) of varying size. As expected, fluctuations in nitrogen availability drastically remodeled the transcriptome, with over half of the genes in the genome found to be responsive to N-replete (RT102) or N-deplete (RT184) status (Fig. 1a, Supplementary Data 1). In the same experiment, we were able to detect protein abundance for 6299/12,178 genes, which we compared to the transcript-level response. To do this, we standardized expression patterns for each protein, assigned them to 100 proteome clusters (PC1–PC100) of varying size, and evaluated the relationship between these PCs and the transcriptome RTs. Two PCs emerged (PC57, PC47) that were enriched in genes belonging to RT102 and RT184 respectively, and there was strong agreement between expression of the transcriptome and proteome for these genes (RT102xPC57, RT184xPC47, Fig. 1b, Supplementary Fig. 1). Functionally, genes upregulated when nitrogen is available are those involved in photosynthesis and growth, including components of the light-harvesting complex, ribosomal proteins, and key amino acid biosynthesis genes. During N-limitation, components of the proteasome and lysosome and endocytosis and autophagy-related machineries were upregulated, evidence of increased recycling of internal nitrogen. The strong impact that overall nitrogen status has on gene expression and regulation of these processes is consistent with previous studies on nitrogen-replete and nitrogen-deplete states, though none of these studies has evaluated the expression on such short timescales^14,17^.

The design of this experiment provided an opportunity to identify genes with a specific response to nitrate, and transcriptome clustering revealed that a small subset of genes (*n* = 160) belonged to RTs that were more highly expressed on nitrate relative to other nitrogen sources (Fig. 1a). Clustering is well suited to characterizing such patterns across a large dataset, but we aimed to precisely identify genes that are nitrate responsive and therefore developed a complementary metric to quantify the strength of the transcript response specifically to nitrate in this experiment (“Methods”). This metric identified genes that were induced with nitrate addition and repressed on ammonia and nitrite. Using these criteria, we identified a set of genes (*n* = 172) that were nitrate-specific (NS) at the transcript level (Fig. 1c). Of these, 61 genes also belonged to NS RTs and were therefore considered HNS (Fig. 1d). Nitrate assimilation genes, including two nitrate transporters, a putative nitrate vacuolar transporter, and genes of the complete nitrate assimilation pathway, were in the HNS set, along with a gene involved in synthesis of the molybdenum cofactor required by nitrate reductase (NR; Fig. 1c). Other HNS genes comprise an intriguing list of candidate proteins putatively involved in the sensing and regulation of nitrate assimilation or related processes (Supplementary Data 1).

**Fig. 2:**
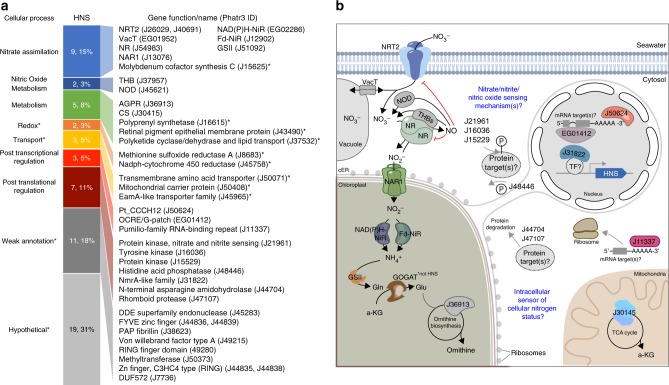
Functional annotations of highly nitrate-sensitive (HNS) gene set and model of nitrate assimilation and regulatory components in *Phaeodactylum tricornutum*. **a** Cellular processes and associated annotation descriptions of the HNS; numbers indicate total genes belonging to each process and percentage of the HNS that process comprises. When possible, the gene name is indicated, otherwise, pFAM descriptions are shown. Full annotations are available in Supplementary Data 3. Phatr3 model IDs are shown for each gene. Asterisk (*) = gene or process is not depicted in **b**. **b** Scheme (after Sanz-Luque et al.^3^) of nitrate assimilation pathway showing HNS genes with putative roles in nitric oxide metabolism (THB, NOD) and posttranscriptional and posttranslational regulation of unknown mRNA and protein targets. The HNS genes of the ornithine biosynthesis pathway and TCA cycle are also depicted. cER chloroplast endoplasmic reticulum

Half of the HNS genes were either unannotated or weakly annotated, but several have conserved domains giving insight into putative roles in nitrate assimilation (Fig. 2). Two of the strongest HNS genes in the HNS gene set were part of the globin superfamily, which are heme-containing proteins that bind oxygen. The first (J37967) is a bacterial-like globin (PF01152) with significant homology to THB1 from *Chlamydomonas reinhardtii* recently found to modulate nitric oxide levels and nitrate reductase activity^18^. The second gene (J45621) contains a globin domain of flavohemoglobins (cd08922) that function predominantly as nitric oxide dioxygenases (NOD). THB1 and NOD convert NO to nitrate, and the strong HNS co-expression of the putative *P. tricornutum* genes with NR points to a similar role in this diatom (Fig. 2b). Some metabolism-related genes, including citrate synthase (CS, J30145) and *N*-acetyl-gamma-glutamyl-phosphate reductase (AGPR, J36913), were HNS, along with a few transporters (Fig. 2). Nitrate sensitivity of these genes may provide some insight into their function during growth on this N source relative to others, though further investigation is needed to confirm this.

A large proportion (16%) of HNS genes has putative roles in regulation of nitrate assimilation, particularly by posttranscriptional or posttranslational regulation (Fig. 2). A single gene from the annotated transcription factors (TFs) in *P. tricornutum*^19^ was identified within the HNS (*Pt_CCCH12*, J50624). The Zn finger CCCH domain (PF00642) it contains is now recognized to mediate protein–protein interactions and protein–RNA interactions. In addition, Pt_CCCH12 contains a YTH domain (PF04146/IPR007275), which binds the 3′-untranslated region (UTR) of mRNA in yeast^20^. This casts doubt on Pt_CCCH12 as a DNA-binding TF; it is more likely to be involved in posttranscriptional regulation. Two other HNS genes were likely to be involved in posttranscriptional regulation; Phatr3_EG01412 contains both G-patch (PF01585) and OCRE (PF17780) domains thought to have a role in RNA binding and splicing respectively, and Phatr3_J11337 has a Pumilio family RNA-binding repeat domain (PF00806). Pumilio is evolutionarily conserved from yeast to humans and plants and is known to regulate aspects of development by controlling mRNA stability and translation through interaction with the 3′-UTR. Several other genes have putative roles in posttranslational regulation, including three protein kinases, a phosphatase, and two genes involved in protein degradation (an N-terminal asparagine amidohydrolase and a rhomboid protease, Fig. 2). An NmrA-like protein (J31822) was also among the HNS set. In fungi, NmrA represses the GATA-type TF AreA responsible for the global response to low N or sub-optimal sources of N including nitrate^5^.

In order to determine whether these HNS genes are homologs of known and/or hypothesized regulatory components from other model organisms (i.e., *C. reinhardtii, Aspergillus nidulans*), we compiled a list of TFs and posttranscriptional and posttranslational regulators in cyanobacteria, yeast, plants, algae, and fungi from the literature and screened the *P. tricornutum* genome for candidate homologs (Supplementary Table 1, “Methods”)^3,5,21–33^. The only putative homolog of these known regulators we could identify was a protein (J50045, RT179) that contained the fungal Zn(2)-Cys(6) binuclear cluster domain (PF00172) also found in the pathway-specific nitrate assimilation TF (nirA) from *A. nidulans*^5,29^. However, this domain is also found in TFs that regulate non-nitrogen-related processes, therefore additional research is needed to confirm whether it has a role in regulating nitrate assimilation in *P. tricornutum*. TFs of the helix-turn-helix type (Pfam clan HTH, CL0123) regulate the expression of nitrate assimilation genes in *Synechococcus* (ntcA) and *Cyanidioschyzon merolae* (CmMYB1)^26,27^. *P. tricornutum* does not have any obvious homologs of ntcA or CmMYB1 but has several helix-turn-helix-type TFs including heat shock factors, which have radiated massively in diatoms^19,34^. It is difficult to narrow down which TFs may be regulators of the nitrate response based on the expression data, since most TFs (including RGQs) were broadly responsive to N-replete or N-deplete status at the transcript level (Supplementary Fig. 2)^35^.

**Fig. 3:**
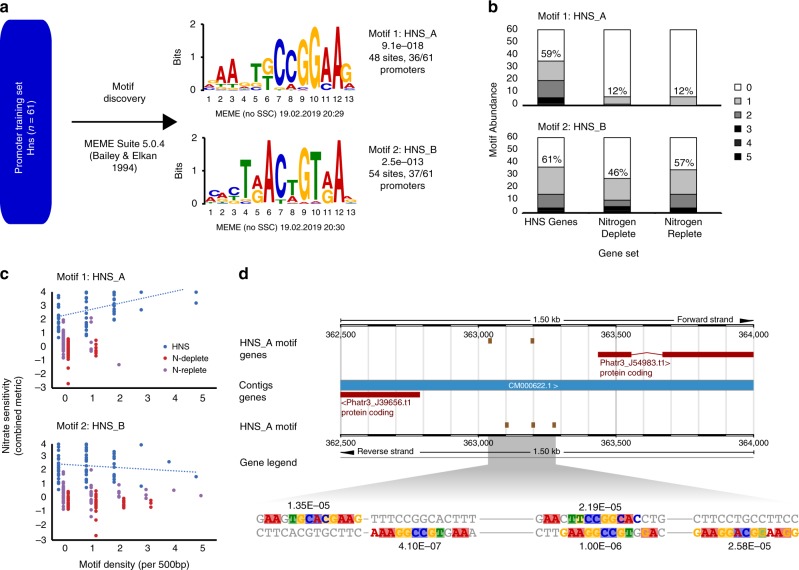
Transcription factor-binding site identification in highly nitrate-sensitive genes. **a** Identification of motifs detected using promoters (500 bp upstream of ORF) of the HNS genes as a training set *E* value (MEME) is shown. **b** Motif abundance in HNS promoters and promoters of nitrogen-deplete and -replete response types (RT184 and RT102). For RT184 and RT102, data show percentages from randomly selected subsets (*n* = 61 genes each RT). **c** Relationship between motif density and nitrate sensitivity metric for HNS and randomly selected subsets of RT184 and RT102. **d** Architecture of the (~647 bp) NR promoter and co-expressed hypothetical J39656, both part of the HNS showing locations and variants of HNS_A motifs. FIMO *p* values are adjacent to motifs. Residues predicted by the motif (>0.5 bit score) are colored, the font shows the actual residue, and background shows predicted residue. Source data are provided as a Source Data file

Though we did not identify strong candidates for TFs that regulate the HNS response from expression data, we used the promoters of the HNS set to identify a motif that could function as a TF-binding site (TFBS). The 500 bp region upstream of genes in the HNS set was the input for MEME, which returned two significant motifs (Fig. 3a, Supplementary Table 2). The first motif (Motif 1, HNS_A) was enriched in the HNS set relative to genes with a general N-deplete or N-replete response, but the second motif (Motif 2, HNS_B) was common in all RTs (Fig. 3b), indicating that it is not involved in regulating the HNS response. Further, there was a higher density of HNS_A motifs in HNS promoters than in other RTs, with a positive relationship between HNS_A density and strength of the HNS response that was not seen for HNS_B (Fig. 3b, c). *NR* and a chloroplast-targeted protein with unknown function, both HNS genes, share five HNS_A motifs in a putative bidirectional promoter (Fig. 3d). We compared our HNS_A motif using TOMTOM to the JASPAR CORE database, a curated set of eukaryotic TFBS, to identify the class of TFs that might bind and regulate this site^36,37^. This returned 38 motifs, 2 of which were highly similar (*p* value <1E–04, Benjamini–Hochberg) to motifs bound by ETS-family TFs from *Drosophila melanogaster* that shared CCGGAAG with HNS_A (Supplementary Data 2). ETS, only found in metazoans and important in development, is a winged-helix-turn-helix TF from the Pfam clan (CL0123) that nitrate regulators from *Synechococcus* and *C. merolae* belong to. This lends strong support to the idea that the nitrate regulator or regulators of diatoms is a helix-turn-helix-type TF, in contrast to the GATA-type, RWP-RK, and Zn-cluster types found in green algal, plant, and fungal models (Supplementary Table 1).

### Configuration, evolution, and function of nitrogen pathways

**Fig. 4:**
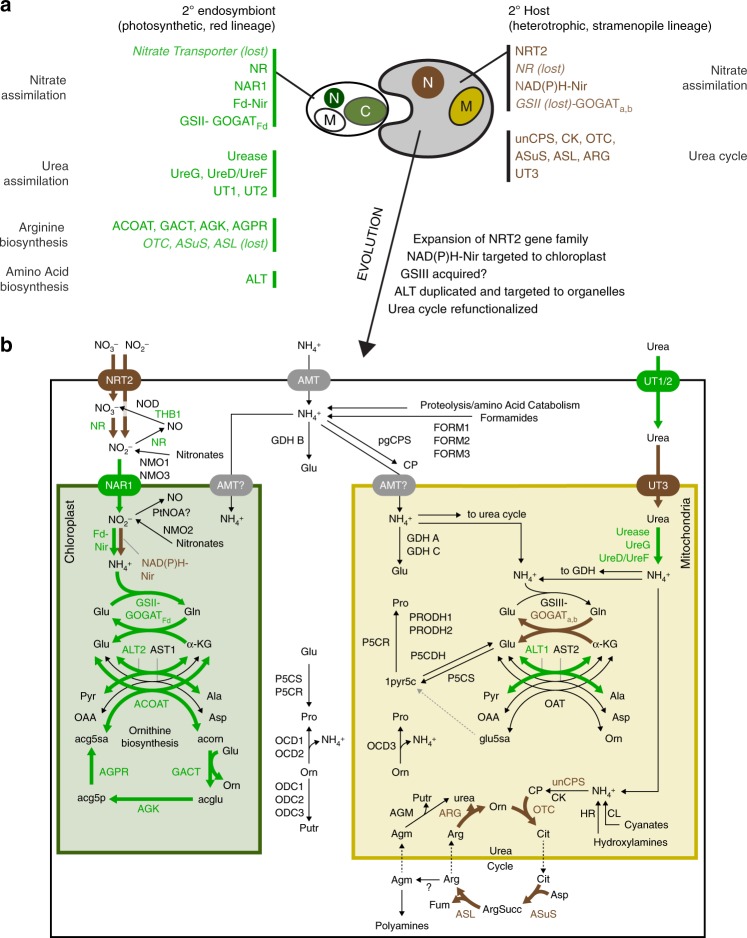
Model of primary nitrogen assimilation and evolution in *P. tricornutum*. **a** Putative origins of nitrogen metabolic genes in extant diatoms identifying components contributed by the endosymbiont or host. **b** Schematic of compartmentation of major nitrogen assimilation, turnover, and links between the connected pathways. Abbreviated gene names are shown in bold; full names and accessions can be found in Supplementary Data 3

The overall configuration, evolutionary origins, and function of diatom nitrogen metabolic networks, as compared to that from other nitrogen model organisms, remain undescribed^9^. To evaluate this more thoroughly, a catalog of genes with putative roles in assimilation of nitrate, urea, and ammonia; nitric oxide metabolism; ornithine biosynthesis; urea cycle; polyamine biosynthesis; and proline metabolism (*n* = 74) was assembled from the *P. tricornutum* genome (Supplementary Data 3). Subcellular protein localizations predicted bioinformatically and confirmed experimentally in selected cases were used to generate an updated model of primary nitrogen metabolism in *P. tricornutum* (Fig. 4). Origins of these genes were inferred through the construction of phylogenies (*n* = 42, Figshare^38^) with the goal of describing features of extant diatom nitrogen metabolism that are similar to those known from plants/algae, animals/fungi, bacteria, or are unique to diatoms. Gene expression of these pathways from the N_short_ experiment was also evaluated to provide insight into the putative functions.

**Fig. 5:**
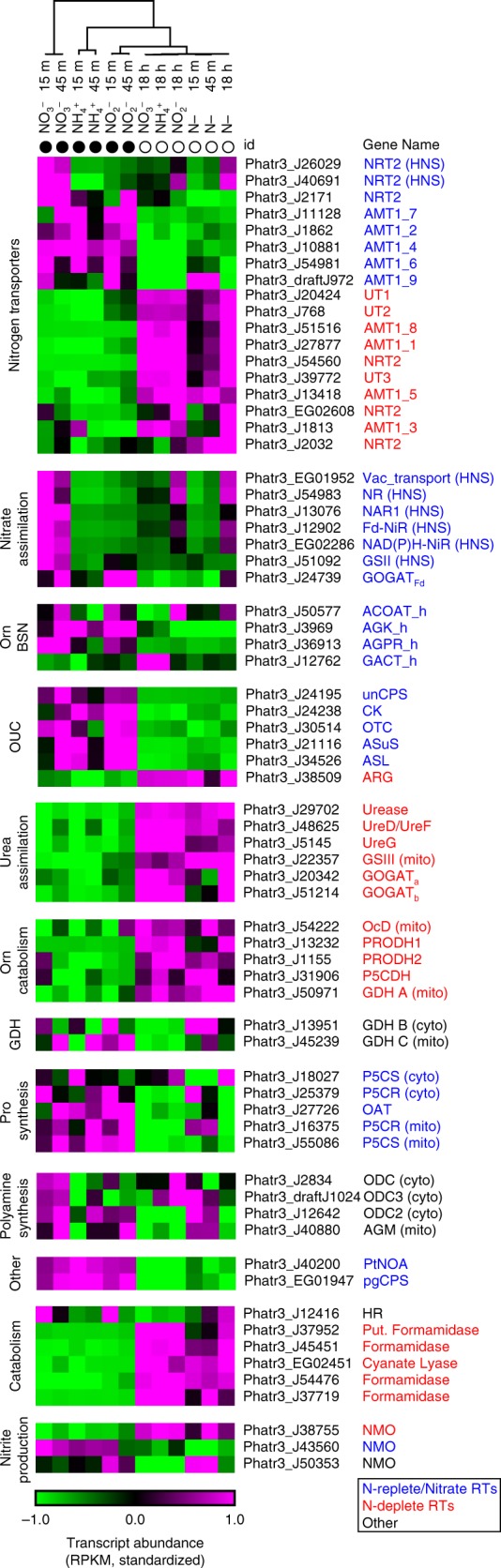
Transcript-level expression of nitrogen assimilation genes and intersecting pathways. Heatmap shows standardized transcript abundance (RPKM) for nitrogen catalog genes, identified with protein ID and gene names, and organized by known or hypothesized pathways. Gene name abbreviations can be found in Supplementary Data 3. Dendrogram shows hierarchical clustering (one-minus Pearson correlation, average linkage) of nitrogen transporter genes only, with the other genes in the same arrangement. Closed circles indicate N-replete time points, open circles show N-deplete time points. Color of gene name indicates whether it was in a nitrate-specific or N-replete response type (blue), an N-deplete response type (red), or other (black). Source data are provided as a Source Data file

Nitrate assimilation pathway genes are largely placed within phototrophic clades indicating that diatoms acquired the ability to assimilate nitrate from the endosymbiont^38^. However, NRT2 and NAD(P)H-NiR clade more closely with sequences from oomycetes than to other secondary endosymbionts, and extant oomycetes have different versions of NR and GSII than diatoms. This indicates that, prior to endosymbiosis, the host possessed a full pathway for assimilation and utilization of nitrate and that host NR and GSII were lost in favor of the endosymbiont versions as diatoms evolved. Host NAD(P)H-NiR was re-targeted to the chloroplast (Supplementary Fig. 3) and host NRT2 remained localized at the outer membrane. After endosymbiosis, NRT2 radiated within the diatom lineage suggesting a period of functional diversification or neofunctionalization after divergence from other stramenopiles^38–40^. Variable expression of NRT2s may reflect specialized uses, perhaps in nitrate affinity (Fig. 5). Regardless of origin, most nitrate assimilation genes are co-expressed (HNS) indicating they have been placed under a shared transcriptional control mechanism in modern diatoms (Fig. 5).

Diatoms assimilate nitrogen from sources other than nitrate including ammonia and urea, growing at comparable rates^41–43^. Ammonia can be imported by one or more of the nine ammonium transporters (AMTs). Like NRT2s, diatom AMTs are monophyletic (except J51516) and have radiated in diatoms but are highly divergent from animal and oomycete sequences^38,39^. Half of the AMTs are upregulated during N-replete states, but some are highly induced by N-limitation like AMT1_1 (J27877), which is the most highly expressed of any of the nitrogen transporters (Fig. 5). *P. tricornutum* has two (recently duplicated) outer membrane-localized urea transporters (UT1, UT2, Supplementary Fig. 4) that clade with sequences from other phototrophs and terrestrial plants, while mitochondrial UT3 is present in only a few extant marine phototrophic lineages and clades with sequences from metazoans^38^. Imported urea is hydrolyzed by urease in the mitochondria (Supplementary Fig. 4). Phylogenetically, urease and accessory proteins are most similar to those found in diverse secondary endosymbionts, indicating that urea hydrolysis was most likely acquired with the endosymbiont^38^. All urea assimilation genes (including transporters) were maximally expressed in nitrogen-limited conditions, indicating that urea transport and hydrolysis is important during N-limited cell states (Fig. 5).

**Fig. 6:**
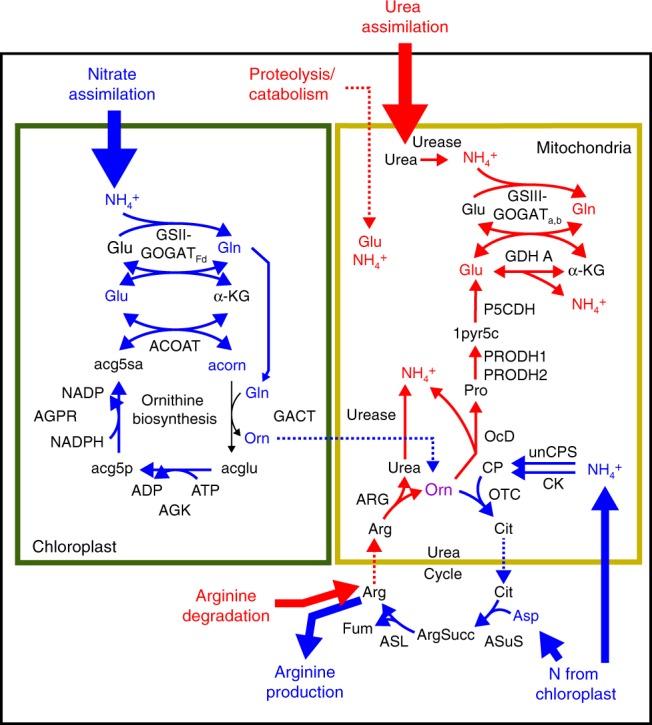
Configuration of organellar GS-GOGAT cycles and split-organelle arginine biosynthesis. Pathway diagram of chloroplast nitrate assimilation and mitochondrial urea assimilation. Pathways are colored to indicate nitrate/N-replete upregulation (blue) or N-deplete upregulation (red). Gene name abbreviations can be found in Supplementary Data 3

The role of the urea cycle across different physiological states is not well understood. In the N_short_ experiment, expression of the full urea cycle was not coordinated; the first several steps were most highly expressed when N was replete while the final step, catalyzed by arginase, had the opposite pattern (Fig. 5). Functionally, this indicates that urea cycle is divided into anabolic and catabolic segments, whereby the first several steps (including unCPS) are upregulated by nitrogen availability, suggesting that they are utilized for the synthesis of arginine or arginine derivatives and the final step is used for arginine catabolism (Fig. 6). Urease hydrolyzes urea produced by arginase into ammonium that can be re-incorporated by GSIII-GOGAT_a,b_ and/or GDH A for cellular redistribution. During catabolism, re-incorporation of arginase-produced urea by the urea cycle would represent a futile cycle (i.e., ammonium derived from arginine catabolism is not likely to be used for arginine synthesis). Ornithine produced by arginase can also be catabolized to glutamate and/or ammonium by a pathway comprised of ornithine cyclodeaminase, proline dehydrogenase, and 1-pyrroline-5-carboxylate dehydrogenase and GDH A (Figs. 5 and 6). This data supports the role of the urea cycle as a repackaging and redistribution hub and identifies the downstream pathways needed to fully recycle nitrogen from cellular arginine^9^.

The second GS-GOGAT cycle (GSIII-GOGAT_a,b_) found in diatom mitochondria appears to be specialized to recover recycled nitrogen and/or assimilate urea. Both GOGAT subunits (GOGAT_a_, GOGAT_b_) clade with oomycetes and have clear mitochondrial targeting signals, suggesting that they were maintained from exosymbiont mitochondria. However, GSIII is similar to sequences found in a range of diverse marine phototrophs, including marine greens (*Ostreococcus*, *Micromonas*), other secondary endosymbionts with a red-algal-derived plastid (haptophytes, crytophytes), and to secondary endosymbionts with a green-algal-derived plastid (euglenophytes). Heterotrophic relatives of diatoms do lack GSIII, therefore it appears that GSIII (like urea assimilation) was acquired from the endosymbiont.

For the urea cycle to function as an arginine biosynthetic pathway during anabolic cellular states, there must be continued nitrogen inputs in the form of ornithine, ammonia, and aspartate (Fig. 6). On nitrate, the chloroplast must be the initial source of these nitrogen inputs, but the metabolic networks, reactions, and transport linking nitrogen metabolism between the plastid and urea cycle are not well described. Ornithine could be supplied directly by the chloroplast, since diatoms have an endosymbiont-derived chloroplast ornithine biosynthesis pathway analogous to one found in plants^15,38^. The ornithine biosynthesis pathway, like the urea cycle, is mostly upregulated during N-replete conditions (with the exception of GACT) indicating that it is anabolic (Fig. 5). Further, the HNS expression of AGPR suggests that plastid ornithine production may be particularly important during growth on nitrate. The diatom arginine biosynthesis pathway is apparently configured differently than what is known from plants. In plants, the entire pathway is in the chloroplast^44^, and in diatoms it split across the chloroplast and mitochondria.

Expression of other nitrogen catalog genes with putative and/or poorly understood roles was evaluated for evidence of function in the nitrogen metabolism and biology of diatoms (Fig. 5). Cytosolic and mitochondrial proline biosynthesis genes and pgCPS were upregulated in N-replete cell states, suggesting that enhanced proline and pyrimidine synthesis occurs under these conditions, but genes with putative roles in polyamine biosynthesis were not consistently dynamically regulated at the transcript level during shifting N. Several nitrogen catalog genes strongly upregulated during nitrogen limited cell states are putatively involved in the catabolism of compounds such as nitronates (NMO1), formamides (FORM1, FORM2, FORM3), and cyanates in reactions that could scavenge nitrogen from uncharacterized sources (Fig. 5). The exact substrates of these genes are unknown, but generally they act on organic molecules to release ammonium or nitrite. These catabolic processes could be an important part of the overall nitrogen budget in these organisms.

### Influence of nitrogen source on pathway utilization

To test hypotheses about intracellular metabolite fluxes from pathway localization and expression data, specifically regarding function of the split-organelle arginine biosynthesis pathway and conditional operation of the organellar GS-GOGAT cycles, we used a combination of biochemical composition data, fluxomics, and genome-scale flux balance analysis. First, whole-cell carbon and nitrogen quotas were quantified in *P. tricornutum* cultures in nitrogen-sufficient, early-stage nitrogen starvation, and in nitrogen-deficient states to establish a range of variation in biochemical composition (Supplementary Fig. 5). Cellular carbon quotas varied 10-fold, ranging between ~10^2^ and 10^3^ femtomoles per cell, consistent with the accumulation of carbon-rich lipids induced during nitrogen deprivation^15^. Nitrogen quotas varied less overall and were generally between 60 and 100 femtomoles per cell. Shifts in C:N were largely driven by increases in cellular carbon quotas as cultures exhaust available nitrogen. Though the total nitrogen cell quota did not vary substantially, metabolite levels were highly dynamic on short timescales in the N_short_ experiment (Supplementary Fig. 5). Most nitrogenous metabolites detected were more abundant when nitrogen was replete, including glutamine. Glutamine and α-ketoglutarate (α-KG) levels were highly correlated. α-Ketoisocaproic acid, a product of leucine degradation, was one of the few metabolites that were elevated when nitrogen was limiting.

Short-term fluctuations in metabolite levels indicate that nitrogen pathway usage is highly flexible, but pathway utilization under variable nitrogen scenarios is unknown. We investigated further by comparing the impact of a nitrogen source assimilated in the chloroplast (nitrate) or mitochondria (urea) on metabolite composition and metabolic pathway usage with both stable isotope tracking experiments and in silico metabolic modeling.

**Fig. 7:**
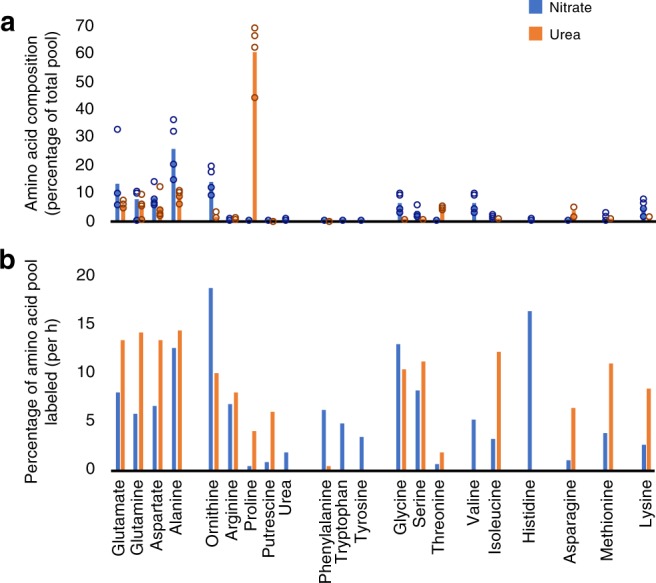
Nitrogenous metabolite composition and labeling rate in *P. tricornutum* grown on nitrate and urea sources. **a** Cellular composition of nitrogenous metabolites expressed as a percentage of the total for the metabolites shown. Metabolites are grouped according to biosynthetic pathways. Data represent the average of four samples taken across the labeling period and are overlaid with individual data points. **b** ^15^N-labeling rate, as the percentage of the metabolite pool that becomes labeled per hour calculated from the 3 h measurement. Source data are provided as a Source Data file

In flux experiments, cultures acclimated to growth on nitrate or urea were given a dose of ^15^N-labeled compound, respective to condition. Metabolite composition and ^15^N content of various amino acid pools was examined at 0, 1, 3, and 8 h following introduction of the ^15^N label during the illuminated phase of normal batch culture. Metabolite composition varied on different nitrogen sources (Fig. 7a). Glutamate, glutamine, aspartate, and alanine labeled quickly on both substrates as full sets of enzymes needed to catalyze their synthesis are found in each organelle (Fig. 7b). Phenylalanine, tryptophan, and tyrosine, aromatic amino acids synthesized in the chloroplast, are nearly exclusively labeled on nitrate indicating that their biosynthesis relies exclusively on glutamate produced in this organelle (Fig. 7b). In contrast, proline and putrescine are nearly exclusively labeled on urea as an N source. Both proline and putrescine are derived from ornithine, which is produced in the chloroplast or in the urea cycle.

The primary pathway that supplies the cell with ornithine is not currently known, but expression data indicate that the chloroplast supplies it during growth on nitrate (Fig. 5). Consistent with this, ornithine was one of the most abundant and the most rapidly labeled nitrogenous metabolite produced on nitrate (Fig. 7). The chloroplast lacks enzymes for ornithine utilization; the enzymes that utilize ornithine (OCD, ODC, OTC, OAT) are in the cytosol and mitochondria making it likely that ornithine is exported from the chloroplast. ^15^N from nitrate is detected in arginine, consistent with chloroplast-derived nitrogen as a substrate for the urea cycle-mediated arginine biosynthesis. Taken together, this is strong experimental support in support for the split-organelle arginine biosynthesis pathway, at least during growth on nitrate.

Overall, differences were observed between both metabolite composition and pathway activity as a function of nitrogen source. To investigate in more detail, we conducted flux balance analysis simulations for growth on nitrate and urea using a genome-scale metabolic network reconstruction of *P. tricornutum*^15^. Several iterations of metabolic flux models were tested, imposing constraints on cross-compartment nitrogen transport (e.g., ammonia) to better understand drivers of organelle-specific nitrogen assimilation. Simulations considered most realistic based on experimental observations (^15^N-labeling patterns) were then compared with respect to overall flux distribution on nitrate vs. urea as the nitrogen source (Supplementary Data 4). Most ammonium produced via nitrate or urea assimilation was assimilated in the respective organelle-specific GS-GOGAT cycles with only modest levels of ammonium flux into the urea cycle simulated on either nitrogen source. Flux through the urea cycle was only to satisfy cellular demand for arginine with none predicted through the arginase reaction. This is not surprising, as the flux predictions only capture the anabolic phase. Ornithine supply varied as a function of nitrogen source. Ornithine was produced by the chloroplast on nitrate and in the cytosol by ornithine cyclodeaminase operating in reverse on urea. Though the simulation could drive the reaction in reverse, we did not consider this to be biologically realistic. Simulations on nitrate, like all other lines of evidence, support the existence of the split-organelle arginine biosynthesis pathway.

**Fig. 8:**
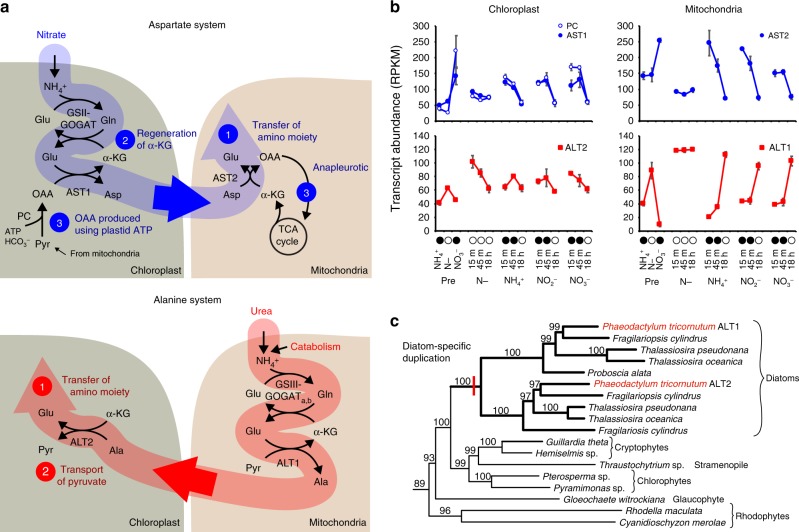
Aspartate and alanine systems for inter-organellar nitrogen and carbon skeleton transport. **a** Localization of nitrogen assimilation pathways and configuration of aspartate and alanine systems across organelles. Gene name abbreviations can be found in Supplementary Data 3 and flux values can be found in Supplementary Data 4. **b** Transcript-level expression of aspartate and alanine system genes from the N_short_ experiment shown with standard deviation. Closed circles on *x* axis indicate N-replete time points, open circles show N-deplete time points. **c** Partial phylogeny of diatom alanine transaminase (ALT) with taxonomic labels. The full tree and ALT sequence accessions can be found in Smith et al.^38^. Source data are provided as a Source Data file

Nitrate assimilation by chloroplast GSII-GOGAT_Fd_ and urea assimilation by mitochondrial GSIII-GOGAT_a,b_ produces glutamine and glutamate, which must be exported from the organelles to satisfy nitrogen demands elsewhere in the cell. Flux simulations predict very little transport of glutamine or glutamate directly relative to alanine and aspartate (Fig. 8). On nitrate, the chloroplast exports aspartate produced by aspartate transaminase (AST1) from glutamate. Once in the mitochondria, glutamate can be produced from aspartate by a corresponding transaminase (AST2). Effectively, this system shuttles nitrogen between the chloroplast and mitochondria on an oxaloacetate (OAA) carbon skeleton. In contrast, nitrogen assimilated from urea is converted to alanine in the mitochondria (alanine transaminase 1 (ALT1)) and transported to the chloroplast where glutamate is regenerated by chloroplast alanine transaminase (ALT2). This system moves nitrogen between organelles on a pyruvate carbon skeleton. The significance of transporting amino moieties between organelles on aspartate and alanine only becomes clearer when considering the larger metabolic network that functions to supply carbon skeleton and balance cellular energetics across organelles.

To sustain nitrate assimilation in the chloroplast, there must be a non-exhausted supply of α-KG. Plastid-localized proteins are capable of direct production of α-KG from photosynthetically fixed carbon, but the reaction kinetics are unfavorable when illuminated. To make α-KG under these conditions, photosynthetically fixed carbon (glyceraldehyde phosphate (GAP)) must first be exported to the mitochondria where it enters glycolysis and is returned to the plastid as pyruvate. Pyruvate can then be converted to OAA by plastid pyruvate carboxylase and transaminated to aspartate (AST1). Aspartate is exported to the mitochondria where AST2 makes glutamate. This aspartate system accomplishes three essential tasks. First, an amino moiety is transferred from the chloroplast to the mitochondria. Second, α-KG is regenerated through aspartate synthesis, and finally, a 4C carbon skeleton (OAA) is returned to the mitochondria in exchange for the 3C skeleton (pyruvate) the chloroplast received. By returning OAA for pyruvate, the cell uses chloroplast energy (from light reactions) to power anapleurotic production of OAA required to sustain the TCA cycle.

Simulated metabolic pathway usage during growth on urea is quite different. Simulations confirm that the TCA cycle supplies sufficient carbon skeletons; however, urea assimilation via GSIII-GOGAT_a,b_ imposes an energetic burden on mitochondria. To compensate, more GAP is imported and glycolysis/respiration is increased in mitochondria. The question remains, why use an alanine system instead of the aspartate system to transport nitrogen to the chloroplast? The chloroplast has a greater demand for pyruvate, which is a more flexible intermediate than OAA. By delivering amino moieties on a pyruvate skeleton (alanine), the mitochondria satisfy the chloroplast demand for pyruvate and do not oversupply a metabolite the plastid does not utilize as readily.

Flux simulations highlight previously unknown and apparently essential roles of AST and ALT systems in cellular coordination of carbon fixation, mitochondrial glycolysis, and nitrogen assimilation across organelles in diatoms. To gain additional insight into the roles of these systems, we evaluated transcript abundance data from the N_short_ experiment. Though the N_short_ experiment did not include a urea condition, the nitrogen-limited time points induced genes involved in urea assimilation, therefore we considered this conditional expression to not only represent overall catabolic conditions but also as a proxy for growth on urea. Consistent with their roles in flux simulations, genes of the aspartate system, including plastid pyruvate carboxylase, were upregulated during nitrogen-replete conditions, and genes of the alanine system were upregulated during nitrogen-deplete conditions (Fig. 8b). The importance of these genes at the intersection of carbon and nitrogen metabolism across the major energetic organelles raises questions about their origins during diatom evolution, particularly surrounding how they may have come together for their current configuration and function.

AST and ALT are central to cellular metabolism, therefore the host and endosymbiont likely possessed distinct isoforms prior to endosymbiosis. The mitochondrial and chloroplast isoforms of ASTs are distinct from one another, but their phylogeny is difficult to interpret, and ultimately the origins of these genes remain unclear; however, the ALT phylogeny is clearer. Extant *P. tricornutum* ALTs belong to a clade containing other secondary endosymbionts (cryptophytes) and genes from the green, red, and glaucophyte archaeplastidal lineages, which is strong evidence for an endosymbiont origin (Fig. 8c). Further, the mitochondrial and chloroplast versions are the result of a diatom-specific duplication. We expanded our analysis to include additional diatom sequences from the Marine Microbial Eukaryote Transcriptome Sequencing Project (Supplementary Fig. 6)^45^. Targeting predictions yield strong support for mitochondrial ALT2, with signal peptides often detected for ALT1. This within-taxon duplication and retargeting suggests that diatoms established the ALT system after plastid acquisition to facilitate delivery of nitrogen and carbon from the mitochondria to the chloroplast during nitrogen-limited cell states.

## Discussion

In this study, we combined a variety of complementary approaches collectively aimed at developing a better understanding of the landscape of nitrogen assimilation and its regulation in diatoms, using *P. tricornutum* as a model. We identified a set of genes that are highly sensitive to nitrate (HNS) that include known homologs of regulatory proteins from algal and fungal models and several other putative posttranscriptional and posttranslational regulators. We also identified a putative TFBS, contributing valuable knowledge about the network of proteins and molecules involved in the regulation of nitrate assimilation and providing a framework for future experimental work. A high degree of energetic and metabolic connectivity between the organelles is an emerging paradigm in diatom biology^46–48^. The data we show here illustrate how diatom pathways are configured to integrate nitrogen metabolism and carbon metabolism between the major energy organelles more intimately than what is observed in green lineages. We propose that this tight metabolic integration allows diatoms to efficiently respond to and assimilate episodic pulses of nitrogen in the marine environment, contributing to their ecological dominance in these conditions.

## Methods

### Growth experiments: short-time N response

A short time course experiment (N_short_) was adapted from earlier investigations of nitrate and ammonium uptake in *P. tricornutum*^16,17^. Duplicate 2 L cultures of *P. tricornutum* (CCAP-1055) were grown on artificial seawater medium with f/2 nutrients, trace metals, and vitamins with 880 μM ammonium as the sole nitrogen source and were stirred and bubbled with air with 14:10 light:dark (150 μE m^−2^ s^−1^) at 18 °C. Cells were collected by centrifugation (10 min × 700 × *g*, 18 °C) at mid-exponential phase (~3 × 10^6^ cells mL^−1^), washed (3×, N-free media), and resuspended in N-free media in respective flasks (2 L) for 2 h. Replicate cultures were spiked with nitrate to 150 μM and incubated for 90 min. Cells were collected by centrifugation, washed (3×, N-free media), and material from each replicate independently split into four 800 mL nitrogen treatments ((1) no nitrogen, or [300 μM] of (2) ammonium, (3) nitrite, or (4) nitrate) at a cell density of ~7.5 × 10^5^ cells mL^−1^. Nitrate [300 μM] was selected for the experiment as previous studies have reported that 300 μM nitrate was exhausted from the media by *P. tricornutum* in exponential phase cultures (~2 × 10^−7^ cells mL^−1^) approximately 3 h after addition^16,17,49^. Samples were taken during the pretreatment (at mid-exponential, and after the 2-h N-pretreatment and 90 min nitrate incubations) and at 15 min, 45 min, and 18 h exposure to each nitrogen treatment.

### RNA extraction and sequencing

Samples collected for RNA were pelleted at high speed at 4 °C; pellets were flash frozen in liquid nitrogen and stored at −80 °C. RNA was extracted using Trizol Reagent (Thermo Fisher Scientific); genomic DNA was removed with DNase I (RNase Free DNase set, Qiagen), followed by RNA clean up with the RNeasy Mini Kit (Qiagen). The quality of total RNA quality was measured on the Agilent 2100 Bioanalyzer. RNA samples were then enriched for mRNA using a Dynabeads mRNA Purification Kit (Thermo Fisher Scientific). Four micrograms of total RNA were used to isolate mRNA from each sample. The mRNA quality was also measured on the Agilent Bioanalyzer. Duplicate libraries were constructed using the ScriptSeq v2 RNA-Seq Kit (Illumina), with library quality verified on the Agilent 2100 Bioanalyzer. Libraries were sequenced on the Illumina HiSeq 2000 platform.

Sequence data (*n* = 30 samples, 15 per replicate experiment) were deposited and are available at the NCBI Sequence Read Archive under the identifier PRJNA311568. Data in this manuscript correspond to libraries SRX1922055-65, SRX1922073-76, SRX1922088-98, and SRX1922105-108. A description of sample IDs and accession numbers can be found in Supplementary Table 3.

### Read-mapping and differential expression

Paired-end Illumina sequencing reads (HiSeq 2 × 100) were quality trimmed and mapped to genomic contigs (both chromosome and bd assemblies) of *P. tricornutum* (http://genome.jgi.doe.gov/Phatr2/Phatr2.download.html). Quality trimming of sequence reads was performed using a Perl script to trim all bases from both ends below Q33 on a single base window, with a minimum trimmed sequence length of 30 bases. Sequencing primer and adapter sequence remnants were also identified for any local alignments by BLASTN (*E*-value < 10) of length at least 10 bp on the terminal ends or length at least 15 bp internally, and reads were removed containing these fragments. This resulted in retention of 94.2% of the total reads, with average read lengths of 99.2 and 98.7 bp for reads 1 and 2, respectively. TopHat v.2.0.14 was used for read mapping, with –mate-inner-dist 100, and –mate-std-dev 200 and all other parameters as default. An average of 63.4% of reads mapped to the Pt3 genome (including chromosomes, bottom-drawer contigs, organelles, and the standards added). Downstream analysis was restricted to this conservative set of high quality trimmed (Q33) and mapped reads.

Gene read counts were measured using FeatureCounts (http://subread.sourceforge.net) based on Phatr2 (http://genome.jgi.doe.gov/cgi-bin/browserLoad?db=Phatr2) and Phatr3 (http://protists.ensembl.org/Phaeodactylum_tricornutum/Info/Index) gene models. Differential expression was calculated using EdgeR^50^. EdgeR version 3.10.5 was used for comparing pairwise control (NH4+, pretreatment) and treatment groups (all N-source time points, 15 min, 45 min, 18 h), with calcNormFactors, estimateCommonDisp, estimateTagwiseDisp for normalization, and exactTest for differential expression, with Benjamini–Hochberg adjusted *p* values, and all other parameters as default. In addition, we calculated relative expression values as reads per kilobase of transcript per million mapped reads (RPKM) from gene read counts and gene lengths as defined in the respective gene models.

### Transcriptome RT binning

To identify the most common broad patterns of expression, individual genes were assigned a coded expression pattern using differential expression determined with EdgeR, where 1 = significantly upregulated (fdr < 0.05), 0 = not significant, and −1 = significantly downregulated (fdr < 0.05) for each time point relative to the ammonium pretreatment (531,441 possible combinations). These codes were concatenated across all conditions for a given gene and clustered hierarchically (agglomerative, one-minus Pearson correlation, average linkage) using the Morpheus software (https://software.broadinstitute.org/morpheus/). Transcriptome RTs were defined by cutting the resulting dendrogram to yield 201 clusters (arbitrary) of varying size. Using coded expression patterns (rather than fold-change values or RPKM) allowed for inclusion of statistical significance with the coarse binning of patterns genome wide.

### Proteomics

Protein samples from the N_short_ experiment replicate B were digested, labeled, and analyzed with iTRAQ-based mass spectrometry (MS) at the Pacific Northwest National Laboratory. The MS proteomics data have been deposited to the ProteomeXchange Consortium via the PRIDE partner repository^51^ with the dataset identifier PXD015061. A description of datasets and accessions can be found in Supplementary Table 4.

### Protein digestion and iTRAQ labeling

Samples were dried with a speed vac. Proteins were then solubilized and denatured in 150 µL of 7 M urea, 2 M thiourea, 4% CHAPS, and 5 mM TCEP in 50 mM ammonium bicarbonate. The samples were vortexed and sonicated into solution and then heated at 60 °C for 30 min. Protein concentrations were determined using the Coomassie Plus Protein Assay (Pierce, Rockford, IL) using a bovine serum albumin standard. Afterwards, the denatured samples were diluted tenfold with 50 mM ammonium bicarbonate, pH 8.0. CaCl_2_ was added to a concentration of 2 mM and trypsin (Affymetrix, Santa Clara, CA) was added at a trypsin:sample ratio of 1:50 (w/w). The samples were digested overnight at 37 °C and then were alkylated with chloroacetamide at a concentration of 5 mM in the dark at 37 °C for 30 min. The peptides were desalted using first an SCX SPE (SUPELCO Supelclean, 100 mg) using 10 mM ammonium formate, pH 3.0, 25% acetonitrile, to wash the peptides, and a 80:15:5 methanol:water:ammonium hydroxide, to elute peptides. The SCX SPE removes the detergent (i.e., CHAPS) but the ammonium salts still need to be removed before iTRAQ labeling. C-18 SPE columns (SUPELCO Discovery, 50 mg) were then employed to remove the salts, using a 0.1% trifluoroacetic acid (TFA) in nanopure water to wash the peptides and 80% acetonitrile and 0.1% TFA in water to elute the peptides. Peptides were then quantified using a BCA assay (Pierce, Rockford IL) with a bovine serum albumin standard. Peptides were labeled with 8-plex iTRAQ (AB Sciex, Redwood City, CA) reagents as described below. Thirty micrograms of each peptide sample was placed in a new tube and dried down. Thirteen micrograms of dissolution buffer (iTRAQ Buffer Kit) was added to each and the sample was vortexed into solution and then was centrifuged briefly to draw sample to the bottom of the tube. The iTRAQ reagent (10 µL) was diluted further with isopropanol (35 µL) and the reagent was then added to each sample. The reaction was carried out at room temperature for 2 h. In all, 50 mM ammonium bicarbonate (200 µL) was added to quench each reaction tube. After 1 h, the contents from all iTRAQ channel reactions were added to one tube and then the sample was vortexed and dried down in a speed vac.

### Peptide fractionation and proteome sample preparation

Labeled peptides were separated using an off-line high pH (pH 10) reversed-phase (RP) separation with an XBridge C18 column from Waters (250 mm × 4.6 mm column containing 5 µm particles and a 4.6 mm × 20 mm guard column) using an Agilent 1200 HPLC System. The sample loaded onto the C18 column was washed for 15 min with Solvent A (10 mM ammonium formate, adjusted to pH 10 with ammonium hydroxide). The LC gradient started with a linear increase of Solvent B (10 mM ammonium formate, pH 10, 90% acetonitrile) to 5% over 10 min, then a linear increase to 45% Solvent B over 65 min, then a linear increase to 100% Solvent B over 15 min, then Solvent B was held at 100% for 10 min, and then was dropped to 0% Solvent B and was held at 100% Solvent A for 20 min. The flow rate was 0.5 mL min^−1^. A total of 48 fractions were collected into a 96-well plate throughout the LC gradient. The high pH RP fractions were then combined into 12 fractions using multiple fraction concatenation^52^. Peptide fractions were dried down and resuspended in nanopure water at a concentration of 0.075 µg µL^−1^ for MS analysis using an LTQ-Orbitrap-Velos MS (Thermo Scientific) system described below.

### MS-based analysis of samples

All peptide samples were analyzed using an automated home-built constant flow nano LC system (Agilent) coupled to an LTQ Orbitrap Velos mass spectrometer (Thermo Fisher Scientific). Electrospray emitters were custom made using 150 µm o.d. × 20 µm o.d. × 20 µm i.d. chemically etched fused silica^53^. An on-line 4 cm × 360 µm o.d. × 150 µm i.d. fused silica capillary analytical column (3 µm Jupiter C18) was used. Mobile phases consisted of 0.1% formic acid in water (A) and 0.1% formic acid acetonitrile (B) operated at 300 nL min^−1^ with a gradient profile as follows (min:%B): 0:5, 2:8, 20:12, 75:35, 97:60, 100:85.

The LTQ Orbitrap Velos mass spectrometer was operated in the data-dependent mode acquiring higher-energy collisional dissociation (HCD) scans (*R* = 7500, 5 × 10^4^ target ions) after each full MS scan (*R* = 30,000, 3 × 10^6^ target ions) for the top ten most abundant ions within the mass range of 300–1800 *m*/*z*. An isolation window of 2.5 Th was used to isolate ions prior to HCD. All HCD scans used normalized collision energy of 45 and maximum injection time of 1000 ms. The dynamic exclusion time was set to 60 s and charge state screening was enabled to reject unassigned and singly charged ions.

### Peptide identification and quantification

For peptide identification, MS/MS spectra were searched against a decoy *P. tricornutum* protein database provided by Andy Allen using SEQUEST^54^. Search parameters included: no enzyme specificity for proteome data and trypsin enzyme specificity with a maximum of two missed cleaves, ±50 ppm precursor mass tolerance, ±0.05 Da product mass tolerance, and carbamidomethylation of cysteines and iTRAQ labeling of lysines and peptide N-termini as fixed modifications. Allowed variable modifications were oxidation of methionine residues. MS-GF+ spectra probability values were also calculated for peptides identified from SEQUEST searches^55^. Measured mass accuracy and MS-GF spectra probability were used to filter identified peptides to <0.4% false discovery rate (FDR) at spectrum level and <1% FDR at peptide level using the decoy approach. iTRAQ reporter ions were extracted using the MASIC software^56^ within 10 ppm mass tolerance of each expected iTRAQ reporter ion from each MS/MS spectrum.

### Protein abundance value determination

Relative abundances of peptides were determined using iTRAQ reporter ion intensity ratios from each MS/MS spectrum. Individual peptide intensity values were determined by dividing the base peak intensity by the relative ratio associated with each reporter ion. All peptide values were then transformed into log2 values. Log2 abundance values from multiple scans of the same peptide were consolidated into a single peptide value by utilizing the Rrollup function in the proteomic software Inferno (http://ashokapol.github.io/Inferno/). The Rrollup function works by taking log2-transformed data and identifying the peptide scan that has the most presence and greatest abundance across all reporter ion channels. All peptide scans resulting from the same peptide are scaled to the most present and abundant peptide and the final single peptide abundance values used represent the median of the scaled abundances. Then each dataset distribution was further normalized using the central tendency normalization algorithm (which normalizes each proteome dataset to the global population median) available in Inferno. Protein abundance values were then determined by using the Rrollup function to convert multiple peptide measurements for a given protein into a single protein value. Peptides unique to only one protein were rolled up together and peptides that could be associated with more than one protein were rolled up into protein groups. Log2-transformed peptide abundances were then mean centered normalized in Excel across all iTRAQ runs associated with the project.

### Proteome clustering

Protein abundance was standardized first within treatment and then across all nitrogen treatments. These standardized abundance profiles were clustered with the Morpheus software (https://software.broadinstitute.org/morpheus, K-means, one-minus Pearson correlation) into 100 clusters. PCs were surveyed for enrichment of transcriptome RTs.

### Nitrate sensitivity and HNS genes

RPKM values were used to quantify the strength of the transcript-level response of each gene to nitrate over N sources, adding a requirement that genes were NH_4_^+^ repressed. Replicate experiments were averaged, and genes were further considered if they had an average RPKM > 5 and RPKM at NO_3_^−^ pretreatment > RPKM at N-pretreatment (i.e., generally upregulated on an N-source). We then calculated NH_4_^+^ repression at 15 min relative to NO_3_^−^ pretreatment, NO_3_^−^ induction at 15 min relative to NH_4_^+^ at 15 min, and NO_3_^−^ induction at 15 min relative to NO_2_^−^ induction at 15 min by simple subtraction and standardization (calculations in Supplementary Data 1). Genes that were above the 85th percentile for each comparison were considered NS. Genes that were both NS and belonged to RTs (from differential expression analysis and clustering) were considered HNS.

### *Cis*-regulatory element identification

Putative promoter sequences (500 bp upstream from the start codon) were extracted from the genome sequence of *Phaeodactylum* (Phatr2_chromosomes_assembly_chromosomes_repeatmasked.fasta) for the HNS gene set (*n* = 61 genes). MEME (v5.0.4) was used to screen for motifs, with default parameters except for the following parameters: Site distribution = anr, Number of motifs = 15, minimum width = 6nt, maximum width = 14nt. FIMO (v5.0.4) was used to screen the genome (chromosomes only). The top motif (HNS_A) was submitted to TOMTOM (v5.0.5) with default parameters and searched against the Eukaryotic DNA, JASPAR CORE, and UniPROBE Mouse database.

### Nitrogen regulator comparative genomics screen

A list of nitrogen metabolic regulators was compiled from the literature (Supplementary Table 1) and used to screen the *P. tricornutum* genome for homologs. The sequences were obtained from Pfams associated with these reference genes were obtained from Uniprot, Joint Genome Institute (JGI, https://genome.jgi.doe.gov/), *C. merolae* Genome Project v3 (http://czon.jp), or the National Center for Biotechnology Information (NCBI, https://www.ncbi.nlm.nih.gov). Pfams associated with reference sequences were obtained from Uniprot or by de novo search in the Pfam 32.0 database. These Pfams were searched in the available annotations for *P. tricornutum* gene models (Phatr3). We also searched for putative homologs using tBLASTn (blast protein vs. translated nucleotide) against the Phatr2 genome with an *E*-value cutoff of 1.0*E*−3, word size = 3, and BLOSUM62 gapped alignment.

### Nitrogen metabolism gene set curation

Annotations from the gene catalog of *P. tricornutum* (http://protists.ensembl.org/Phaeodactylum_tricornutum/) were searched for genes with known and hypothesized roles in nitrogen metabolism. Gene annotations were supplemented with TIGRfams and Pfams using Hmmer3, as well as BLASTP to PhyloDB (https://scripps.ucsd.edu/labs/aallen/data), a peptide database that includes *P. tricornutum* and other relevant genomes and transcriptomes, including all Kegg peptides. TMHMM was also run and integrated with HMM and Blast results for transporter annotations. High-throughput subcellular targeting predictions for the entire catalog were obtained using HECTAR^57^. The nitrogen-specific gene catalog was curated manually with regards to orthology, domain construction, and target peptide presence, including manual extensions of gene model start sites based on expressed sequence tag support. Presence of target peptides in the manually curated set were predicted with MitoFates, MitoProt, TargetP 1.1, SignalP 4.1, and ASAFIND^58–62^.

### Cloning and protein localization

Mitochondrial localization of selected proteins was confirmed with confocal microscopy of transgenic diatoms overexpressing native protein–fluorescent protein fusions (Supplementary Figs. 3  4).

For experimentally validated protein localizations of Fd-Nir, NAD(P)H-Nir, UT1, UT3, and Urease, *P. tricornutum* cDNA encoding full-length open-reading frames were PCR-amplified and cloned into a TOPO pENTR vector (Invitrogen). A clone containing an error-free sequence was selected for Gateway recombination (Invitrogen) with a diatom C-terminal YFP pDONR vector^63^. The resulting expression vector was transformed into *P. tricornutum* by particle bombardment^64^. Transformants were then screened by PCR and used for confocal imaging and electron microscopic immunolocalization using an antiserum against green fluorescent protein.

### Phylogenetic trees

Phylogenies were constructed to infer evolutionary origins of the *P. tricornutum* nitrogen gene set. For each *P. tricornutum* copy of nitrogen metabolism-related genes, blastp of BLAST+ package^65^ was used to extract s from an in-house database containing representative sampling of both prokaryotes and eukaryotes with a special focus to marine primary and secondary algae. BlastP was run with *E*-value threshold set to 1E−15 and all other parameters kept default. The datasets were then aligned using L-INS-i iterative refinement algorithm implemented in MAFFT 7^66^. Hypervariable and/or gappy regions were removed using trimAl^67^. Edited alignments were then analyzed using Maximum likelihood (ML) in IQTree 1.6^68^ under the best-fitting model as estimated by ModelFinder^69^ implemented in IQTree. The complete set of phylogenetic trees is published as a fileset on Figshare^38^.

### Growth experiments: nitrogen quota and fluxomics

Large volume (8 L in 20 L carboys) cultures of *P. tricornutum* were grown at 20 °C on a 14:10 light:dark cycle (125 µE m^−2^ s^−1^) cycle with continuous bubbling and stirring until reaching a cell density of 5 × 10^6^ cells mL^−1^. This cell density provides the necessary biomass, but the culture is still in exponential growth phase. The nitrogen source was either nitrate or urea depending on the experiment. The culture was then gently pelleted by centrifugation (10 min × 5000 × *g*), with subsequent resuspension into two duplicate 4 L batches of artificial seawater amended with replete nutrients (f/2) and 100 mM N. In one experiment, the N source was nitrate (100 mM) while in the other it was urea (50 mM). Within the experiments, one 4 L batch was provided with ^14^N label, while the other was provided with ^15^N (99% purity). Samples (250 mL) for metabolites and transcriptomics were collected by rapid centrifugation (5 min × 10,000 × *g*). Samples were collected at the initiation of the experiment and 1, 3, and 8 h after.

### Metabolomics

For both the N_short_ and fluxomics experiments, metabolites were extracted, and derivatization and gas chromatography time-of-flight (GC-TOF)-MS analysis were carried out^70,71^. For this purpose, for each sample, 50 mg fresh powder was used and mixed with 700 μL 100% methanol and 30 μL ribitol (0.2 mg mL^−1^ stock in water) as an internal quantitative standard for the polar phase. After shaking the mix for 15 min at 70 °C, the extract was centrifuged for 10 min at 20,817 × *g*. The supernatant from the centrifuged mixture was then mixed with 375 μL chloroform and 750 μL water. After centrifugation for 15 min at 1699 × *g*, a 150-μL aliquot from the upper polar phase was taken and dried in vacuum. The dried samples were shipped to Golm, Potsdam for metabolic profiling. After derivatization, 1 μL each sample was injected into a GC-TOF-MS system (Pegasus III; Leco). GC was performed using a 30-m MDN-35 column. The injection temperature was 230 °C, and the transfer line and ion source were set at 250 °C. Metabolites were identified through comparison with database entries of authentic MSRI libraries^72^. For absolute quantification of metabolites, calibration curves of different dilutions of authentic standards were evaluated side by side with the samples^73^. Metabolite profiling data are reported following recent recommendations^74^.

### Constraint-based modeling of metabolism

Metabolic reaction fluxes were simulated using the published genome-scale model (GEM) of *P. tricornutum*^15,75^. Assuming a culture mass of 1 g dry weight (gDW), the model was constrained using a maximum O_2_ evolution rate of 10 mmol gDW^−1^ h^−1^ (model reaction EX_o2_e upper bound = 10) and a maintenance energy requirement of 0.33 mmol O_2_ gDW^−1^ h^−1^ (model reaction AOX_m lower bound = 0.33). The metabolite concentrations measured under nitrate and urea growth conditions were reported as nmol mg^−1^ protein. Using the protein fraction of total biomass from the model’s biomass objective function (70%) and the assumed 1 g culture weight, the metabolite concentrations were converted to mmol gDW^−1^. Using the change in metabolite concentration from *t* = 0 to *t* = 1 h, the rate of metabolite pooling or consumption was determined^76^. The rate of proline accumulation under urea-grown conditions was reduced to 1 mmol gDW^−1^ as the measured rate, 6.6 mmol gDW^−1^, was infeasible. Typically, GEM simulations assume a pseudo-steady state where intracellular metabolite concentrations are time invariant:

1 $$S \cdot v = 0$$

where *S* is a matrix representing the reaction stoichiometry and *v* is the reaction flux vector. For metabolites with time course metabolomics data, we set the concentration change equal to the measured pooling or consumption rates calculated above:

2 $$S \cdot v = {\mathrm{d}}x/{\mathrm{d}}t$$

where *x* is the metabolite concentration. To enforce these metabolite constraints, these rates were integrated into the GEM as sources or sinks in a manner similar to the uFBA method^77^. The upper and lower bounds of these reactions were set to the experimentally determined rate ensuring the model only returned solutions consistent with the metabolite dynamics.

Initial simulation results suggested excessive cross-compartment transport of ammonium. Thus we restricted maximum ammonium exchange between the mitochondria and cytosol and the chloroplast and cytosol, reducing it to 0.1 mmol gDW^−1^. There is no evidence of intracellular ammonium transport in diatoms, so the choice to allow or restrict it is somewhat arbitrary. However, without imposing this constraint, ammonium assimilation occurred nearly exclusively in the chloroplast regardless of N source (due to energy supply) resulting in very little flux through mitochondrial pathways. Considering ^15^N data that indicated mitochondria pathway utilization occurs during growth on urea, we considered the unconstrained simulations to be unlikely.

With the model constrained as such, the simulations were performed by maximizing growth rate (model reaction bof_c) using the loopless_solution implementation based on the CycleFreeFlux method (10.1093/bioinformatics/btv096) in Cobrapy^78^ using the default solver.

### Reporting summary

Further information on research design is available in the Nature Research Reporting Summary linked to this article.

## Supplementary information

Supplementary Information

Description of Additional Supplementary Files

Supplementary Data 1

Supplementary Data 2

Supplementary Data 3

Supplementary Data 4

Reporting Summary

Source Data

## Data Availability

Nucleotide sequence data generated during this study are available at the NCBI Sequence Read Archive (SRA) under the identifier PRJNA311568. Data in this manuscript correspond to libraries SRX1922055-65, SRX1922073-76, SRX1922088-98, and SRX1922105-108. A description of sample IDs and accessions can be found in Supplementary Table 3. Mass spectrometric proteomics data have been deposited to the ProteomeXchange Consortium via the PRIDE partner repository^51^ with the dataset identifier PXD015061. Phylogenetic trees are publicly available via Figshare. Data underlying Figs. 1a, b, d, 2c, 3b, c, 5, 7a, b, and 8b and Supplementary Figs. 1a, b, 2, 5a–d, and 6 are provided as Source Data files. All other data are available from the corresponding author upon request.
