# Supplementary material for: Evolution and regulation of nitrogen flux through compartmentalized metabolic networks in a marine diatom
Source: Nat Commun. 2019 Oct 7;10:4552. doi: 10.1038/s41467-019-12407-y (PMC6779911; doi:10.1038/s41467-019-12407-y)
Supplement: Supplementary file 1 — Supplementary Information [file 41467_2019_12407_MOESM1_ESM.pdf]

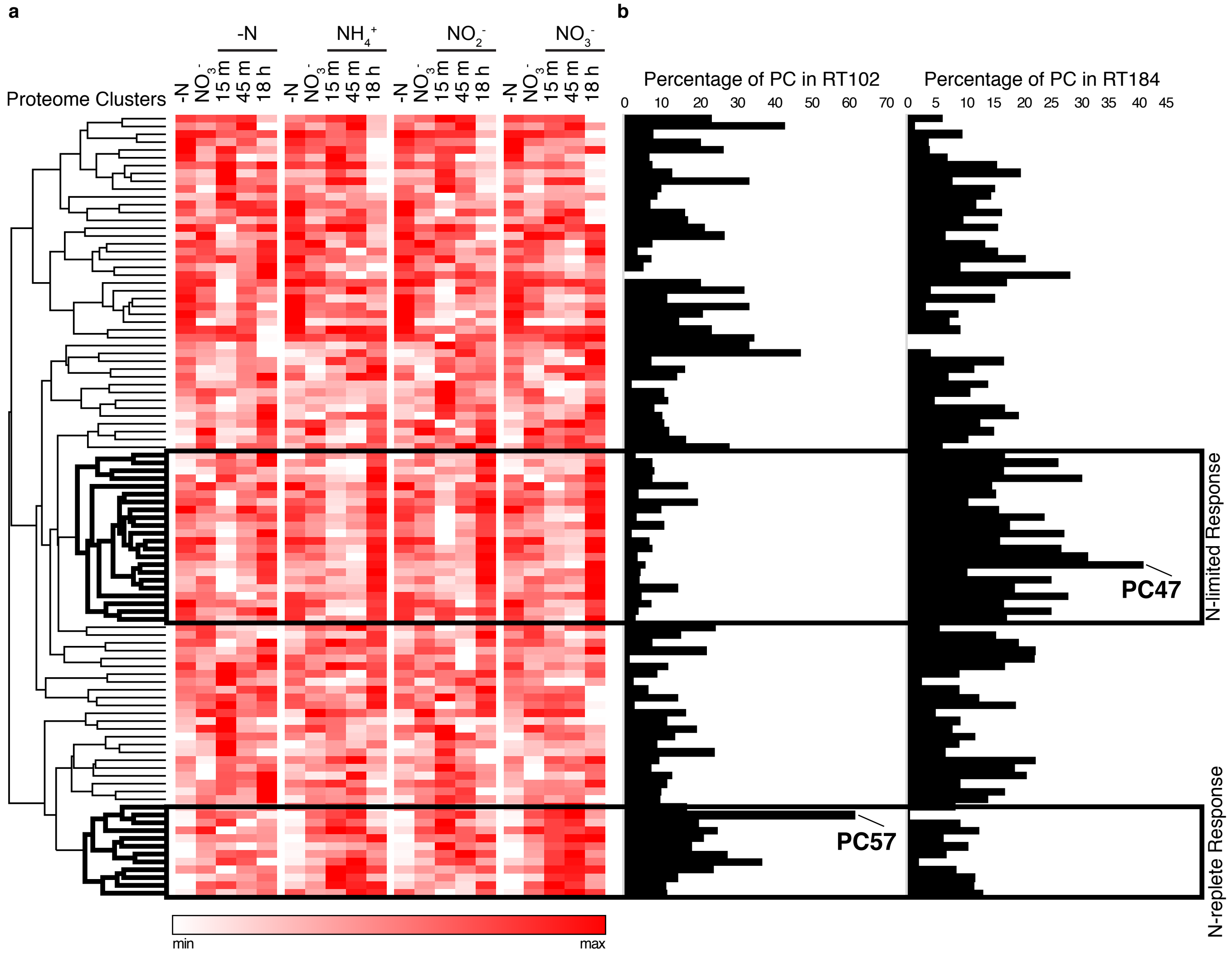

Supplementary Figure 1: Proteome expression and overlap with two major transcriptome response types (RT102, RT184).

(**a**) Heatmap shows average protein expression of all genes contained within each proteome cluster (PC, n = 100), standardized first within treatment and then across all treatments. PC expression averages were then clustered hierarchically, shown by the represented dendogram. (**b**) The proportion of genes within each PC belonging to either of the two major RTs. Generally, PCs that were upregulated in N-replete conditions (i.e. PC57) were represented in RT102, while PCs that were upregulated in N-limited conditions (i.e. PC47) were more represented in RT184. Source data are provided as a Source Data file.

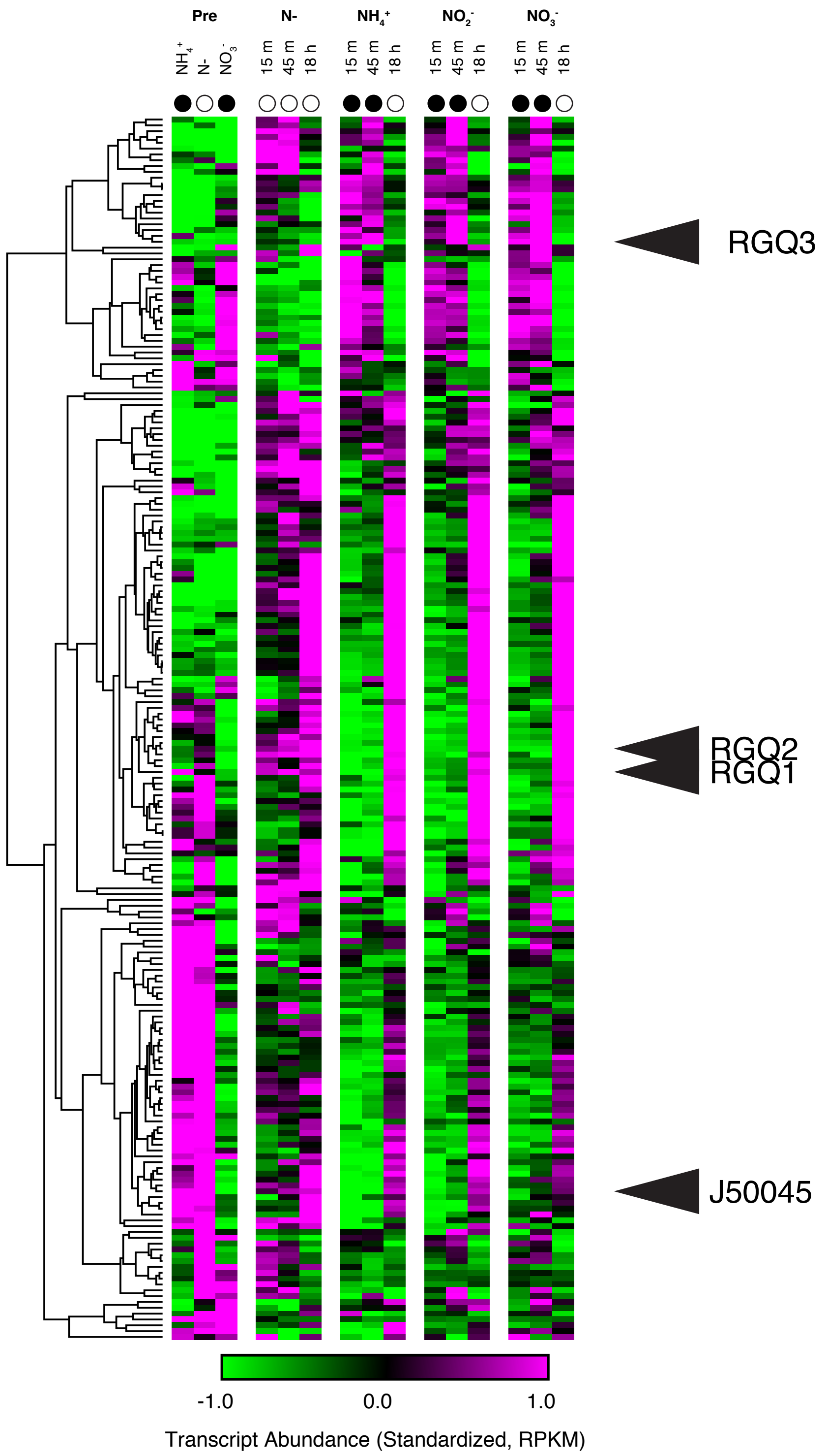

Supplementary Figure 2: Expression of transcription factors in the Nshort experiment.

Heatmap shows standardized transcript abundance of annotated transcription factors in *P. tricornutum* (Rayko et al. 2012). Dendogram shows hierarchical clustering (one-minus pearson, average linkage). Closed circles indicate nitrogen-replete time points, open circles indicate nitrogen-deplete time points. Black arrows indicate transcription factors implicated in the regulation of nitrogen metabolism (Matthijs et al. 2016) and the putative *NirA* homolog identified in *P. tricornutum*. Source data are provided as a Source Data file.

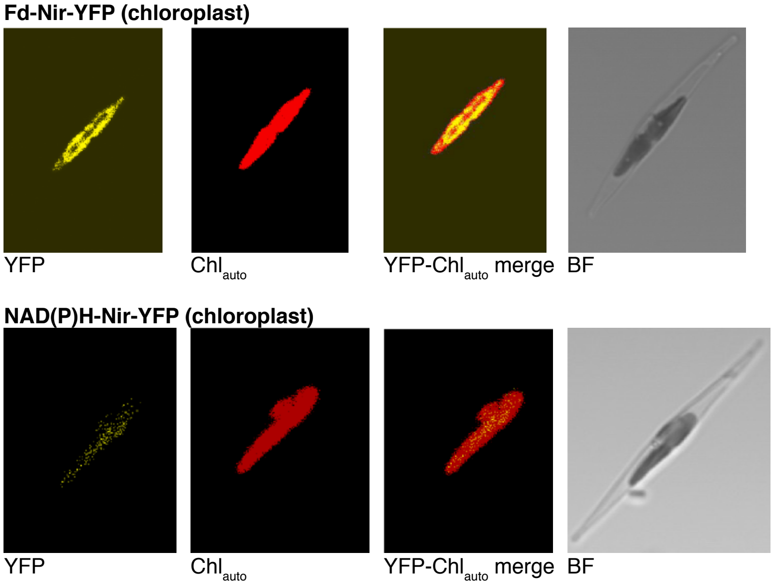

Supplementary Figure 3: Chloroplast localization of Fd-Nir and NAD(P)H-Nir in *P. tricornutum*.

Cells transformed with yellow fluorescent protein (YFP) tagged Fd-Nir and NAD(P)H-Nir were used to examine subcellular localization of both nitrite reductase isozymes in *P. tricornutum*. From left to right, micrographs show YFP fluorescence (yellow), location of chloroplast from detection of chlorophyll autofluorescence (red, Chl$_{auto}$), merged YFP-Chl$_{auto}$, and brightfield (BF).

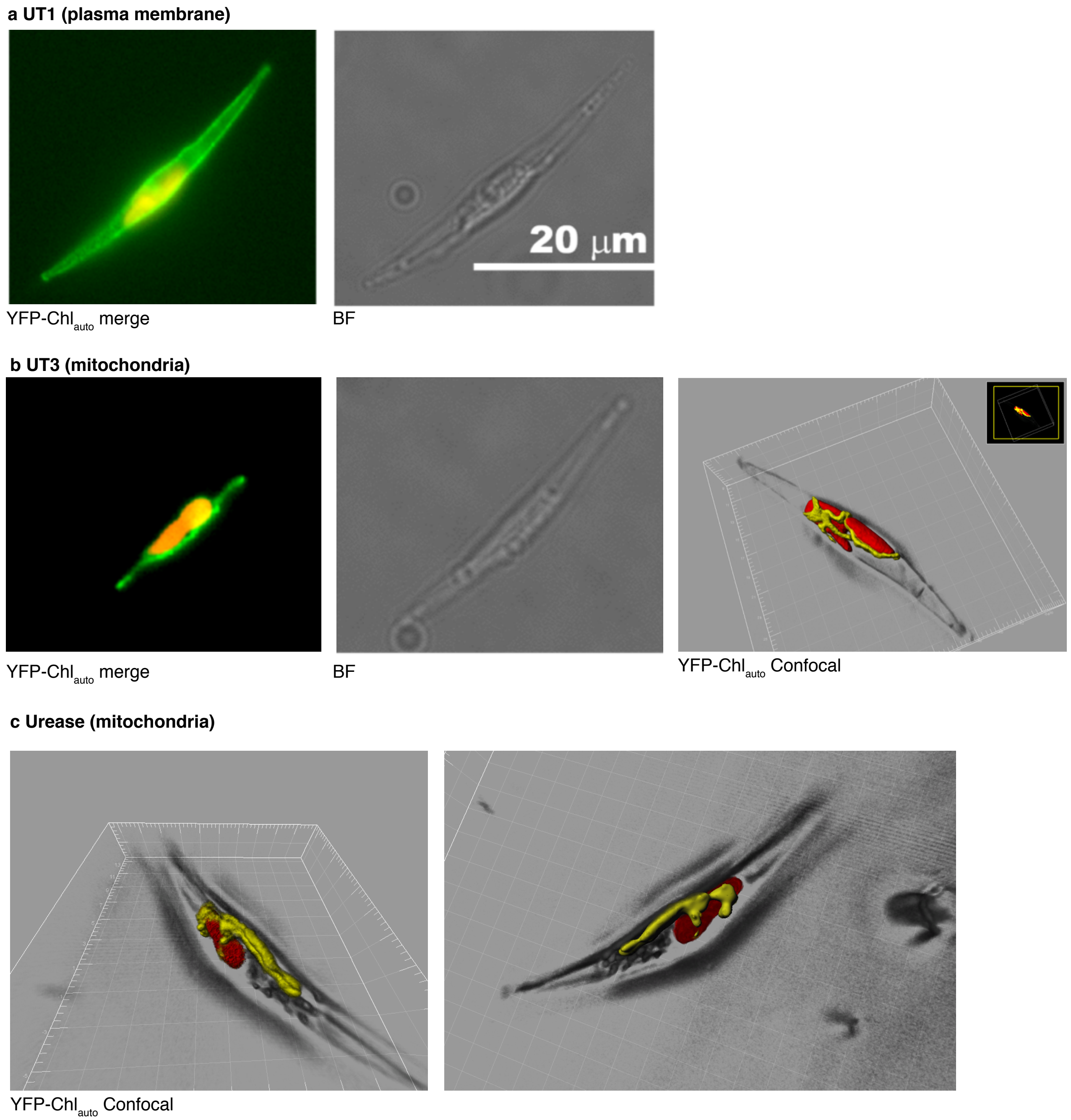

Supplementary Figure 4: Subcellular localization of urea assimilation genes.

Cells transformed with yellow fluorescent protein (YFP) tagged (**a**) YFP-UT1, (**b**) YFP-UT3, and (**c**) YFP-urease were used to examine subcellular localization. (**a,b**) From left to right, micrographs show merged images of YFP fluorescence (yellow) with chlorophyll autofluorescence (red, Chl_{auto}), and brightfield (BF). (**b,c**) Composite 3D confocal image (far right in **b**), showing mitochondrial localization of UT3 (**b**) and urease (**c**).

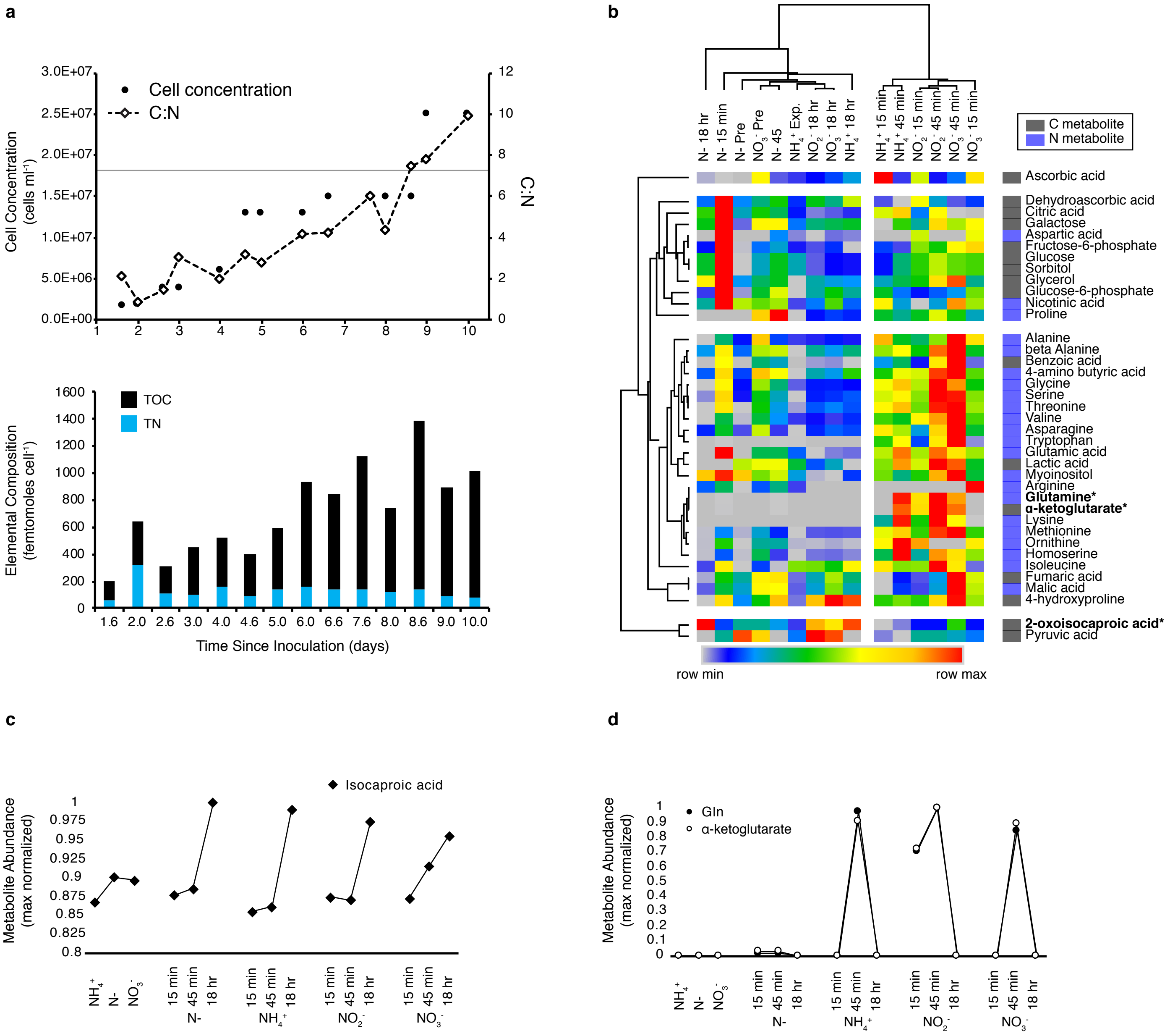

Supplementary Figure 5: Cellular C and N quotas and metabolite composition in *P. tricornutum*.

(**a**) Cell concentration, C:N, and elemental composition of *P. tricornutum* grown in batch culture, TOC: Total organic carbon, TN: Total nitrogen (**b**) Metabolite composition of *P. tricornutum* from N_{short} experiment, max-normalized. Light grey values indicate metabolite was not detected. *Metabolites plotted in (**c**,**d**). (**c**) Isocaproic acid abundance from the N_{short} experiment. (**d**) Glutamine and α-ketoglutarate abundance from the N_{short} experiment. Source data are provided as a Source data file.

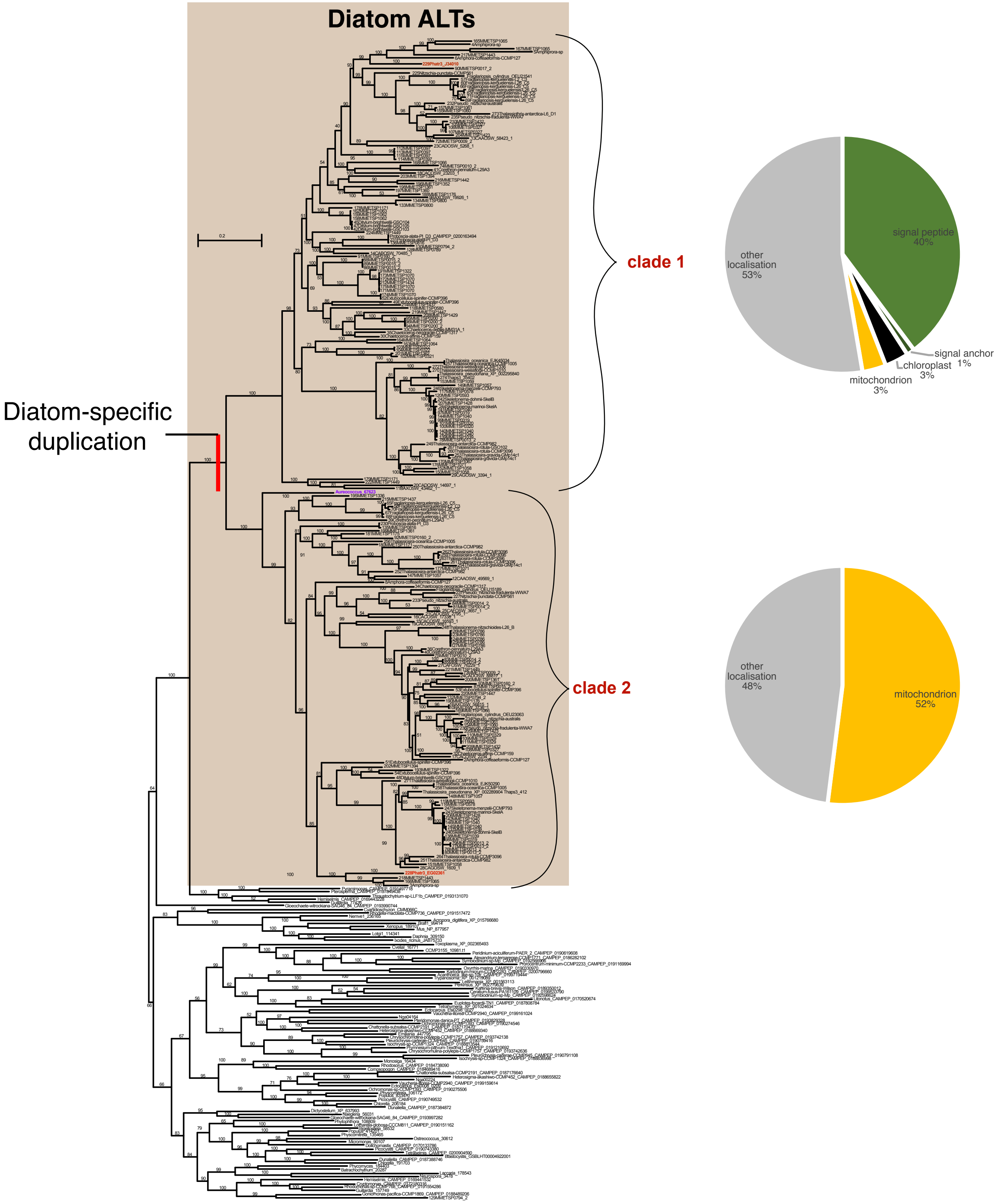

Supplementary Figure 6: Alanine transaminase phylogeny and subcellular localization predictions.

Expanded phylogeny of alanine transaminase (ALT) including diatom sequences from the MMETSP. There are two major diatom ALT clades, which are the result of a diatom-specific ALT duplication (indicated). Sequences from *P. tricornutum* are shown in red. Purple shows placement of an ALT domain trimmed from from an A*ureococcus anophagefferens* sequence, and is likely a contaminant. Pie charts to the right of each clade show the subcellular targeting predictions (TargetP) run on the untrimmed ORFs. Source data are provided as a Source Data file.

Supplementary Table 1: Molecular components of nitrogen response regulation from diverse eukaryotes and candidate homologs in *Phaeodactylum tricornutum*

Eukaryotic genes involved in the cellular response to nitrogen (also including *Synechococcus*) compiled from the literature. Accession sources are specified, including Uniprot, Joint Genome Institute (JGI, https://genome.jgi.doe.gov/), *Cyanidioschyzon merolae* Genome Project v3 (http://czon.jp), or the National Center for Biotechnology Information (NCBI, https://www.ncbi.nlm.nih.gov). Pfams associated with reference sequences were obtained from Uniprot, or by *de novo* search in the Pfam 32.0 database. IDs for candidate homologs in *P. tricornutum* (Phatr3 models) are listed when single-candidate or highly nitrate sensitive (HNS) or nitrate sensitive (NS) genes were identified (see methods). ND = not detected.

Transcriptional Regulators

	Gene Name	Protein	Organism	Accession	PFam	Phatr3 Candidate	Reference(s)
wHTH TF	*ntcA*	Global nitrogen regulator	*Synechococcus sp.*	A5GII0 Uniprot	PF13545 HTHCRP (CL0123 Helix-turn-helix)	ND	Vega-Palas et al. 1992
R2-R3 Myb	*myb1*	MYB-related protein	*Cyanidioschyzon merolae*	CMJ282C Genome	PF00249 myb_DNA binding<br>PF13921 myb_DNA-bind_6 (CL0123 Helix-turn-helix)	n = 21<br>n = 9	Imamura et al. 2009
GATA-Type TF	*Gln3*	Nitrogen regulatory protein GLN3	*Saccharomyces cerevisiae*	P18494 Uniprot	PF00320 GATA (CL0167 Zinc beta ribbon)	ND	Ghaemmaghami et al. 2003
GATA-Type TF	*Gat1*	Transcriptional regulatory protein GAT1	*Saccharomyces cerevisiae*	P43574 Uniprot	PF00320 GATA<br>PF08550 DUF1752	ND<br>ND	Ghaemmaghami et al. 2003
GATA-Type TF	*area*	Nitrogen regulatory protein areA	*Emericella nidulans*	P17429 Uniprot	PF07573 AreA_N<br>PF08550 DUF1752<br>PF00320 GATA	ND<br>ND<br>ND	Marzluf 1997<br>Todd 2016
GATA-Type TF	*nit-2*	Nitrogen catabolic enzyme regulatory protein	*Neurospora crassa*	P19212 Uniprot	PF08550 DUF1752<br>PF00320 GATA	ND<br>ND	Marzluf 1997<br>Todd 2016
RWP-RK TF	*Nit2*	Nitrate assimilation regulatory protein	*Chlamydomonas reinhardtii*	Q2LGZ3 Uniprot	PF02042 RWP-RK (no CL)	ND	Sanz-Luque et al. 2015
RWP-RK TF	*Nlp7*	Protein NLP7	*Arabidopsis thaliana*	Q84TH9 Uniprot	PF02042 RWP-RK	ND	Marchive et al. 2013
Zn-clust TF	*nirA*	Nitrogen assimilation transcription factor nirA	*Emericella nidulans*	P28348 Uniprot	PF04082 Fungal_trans<br>PF00172 Zn_clust	ND<br>n = 1<br>**J50045**	Marzluf 1997<br>Todd 2016

Posttranscriptional/Posttranslational Regulators							
	**Gene**	**Protein**	**Organism**	**Accession**	**PFam**	**Phatr3 Homolog**	**Reference(s)**
nmrA	*nmrA*	Nitrogen metabolite repression protein nmrA	*Emericella nidulans*	Q5AU62 Uniprot	PF05368 NmrA	n = 5<br>**J31822 - HNS**	Marzluf 1997 Todd 2016
	nmr	Nitrogen metabolite repression protein nmr	*Neurospora crassa*	P23762 Uniprot	PF05368 NmrA		Marzluf 1997 Todd 2016
	PcNMRA4	Transcriptional regulator of biotrophy to necrotrophy transition	*Phytophthora capsici*	505845 Phyca11 JGI	PF05368 NmrA		Pham et al. 2018
Gluta-redoxin	*URE2*	Transcriptional regulator URE2	*Saccharomyces cerevisiae*	P23202	PF00043 GST_CTER<br>PF02798 GST_NTER	n = 7<br>n = 3	Coschigano & Magasanik 1991
GC	*CYG56*	Soluble guanylate cyclase	*Chlamydonomas reinhardtii*	B6SE90 Uniprot	PF00211 Guanylate_cyc<br>PF07700 HNOB<br>PF07701 HNOBA	n = 29<br>ND<br>ND	Sanz-Luque et al. 2014
Tandem Zn-finger	*CrNZF1*	Nitrate Zinc Finger 1	*Chlamydomonas reinhardtii*	512330 Chlre4 JGI	PF00642 zf-CCCH	n = 8<br>**J50624 - HNS**	Higuera et al. 2014
Truncated Hemoglobin	*THB1*	Putative truncated hemoglobin	*Chlamydomonas reinhardtii*	A8JAR4 Uniprot	PF01152 Bac_globin	n = 4<br>**J37957 - HNS**	Sanz-Luque et al. 2015
	HaNR2-2/2HbN	Nitrate reductase, truncated hemoglobin fusion	*Heterosigma akashiwo*	JN831656 genbank	PF01152 Bac_globin		Stewart and Coyne 2011
	CsNR2-2/2HbN	Nitrate reductase, truncated hemoglobin fusion	*Chattonella subsalsa*	JN831658 genbank	PF01152 Bac_globin		
Kinase	*MPK6*	Mitogen-activated protein kinase 6	*Arabidopsis thaliana*	Q39026 Uniprot	PF00069 Pkinase	n = 125<br>**J21961 - HNS**<br>**J15229 - HNS**<br>**J5286 - NS**<br>**J46233 - NS**	Wang et al. 2011
PII	*GLB1*	Nitrogen regulatory protein PII	*Chlamydomonas reinhardtii*	A8JI83 Uniprot	PF00543 P-II	ND	Uhrig et al. 2009
ribosome binding GTPase	*NOA1*	NO-associated protein 1, chloroplastic/mitochondrial (note: role in NO production dubious)	*Arabidopsis thaliana*	Q66GP9 Uniprot	PF01926 MMR_HSR1	n = 28<br>**J40200**	Moreau et al. 2008, Vardi et al. 2008

Supplementary Table 2: MEME Output

```
MEME version 5.0.4

ALPHABET= ACGT

strands: + -

Background letter frequencies (from unknown source):
A 0.270 C 0.230 G 0.230 T 0.270

MOTIF 1

log-odds matrix: alength= 4 w= 13 E= 9.1e-018
    31     34     62   -369
   143   -247   -114   -111
   155   -346   -188   -111
   -11    -88    112   -111
 -1223    -88    -47    139
 -1223  -1223    123     76
 -1223    203   -247   -369
   -89    186  -1223   -369
  -369   -247    203  -1223
 -1223  -1223    212  -1223
   163    -47  -1223  -1223
   189  -1223  -1223  -1223
   -52   -188    166   -369


letter-probability matrix: alength= 4 w= 13 nsites= 48 E= 9.1e-
018
0.333333 0.291667 0.354167 0.020833
0.729167 0.041667 0.104167 0.125000
0.791667 0.020833 0.062500 0.125000
0.250000 0.125000 0.500000 0.125000
0.000000 0.125000 0.166667 0.708333
0.000000 0.000000 0.541667 0.458333
0.000000 0.937500 0.041667 0.020833
0.145833 0.833333 0.000000 0.020833
0.020833 0.041667 0.937500 0.000000
0.000000 0.000000 1.000000 0.000000
0.833333 0.166667 0.000000 0.000000
1.000000 0.000000 0.000000 0.000000
0.187500 0.062500 0.729167 0.020833
```

```
MEME version 5.0.4

ALPHABET= ACGT

strands: + -

Background letter frequencies (from unknown source):
A 0.270 C 0.230 G 0.230 T 0.270

MOTIF 2

log-odds matrix: alength= 4 w= 13 E= 2.5e-013
    46    106   -264   -128
    99   -164     17   -106
 -1240    117    -18      4
 -1240   -205   -205    172
    89  -1240    106   -386
   189  -1240  -1240  -1240
 -1240    198  -1240   -154
    -6   -264   -264    131
  -286  -1240    206  -1240
  -286   -363  -1240    181
   104  -1240     95  -1240
   189  -1240  -1240  -1240
    60   -131     69   -106


letter-probability matrix: alength= 4 w= 13 nsites= 54 E= 2.5e-
013
0.370370 0.481481 0.037037 0.111111
0.537037 0.074074 0.259259 0.129630
0.000000 0.518519 0.203704 0.277778
0.000000 0.055556 0.055556 0.888889
0.500000 0.000000 0.481481 0.018519
1.000000 0.000000 0.000000 0.000000
0.000000 0.907407 0.000000 0.092593
0.259259 0.037037 0.037037 0.666667
0.037037 0.000000 0.962963 0.000000
0.037037 0.018519 0.000000 0.944444
0.555556 0.000000 0.444444 0.000000
1.000000 0.000000 0.000000 0.000000
0.407407 0.092593 0.370370 0.129630
```

Supplementary Table 3: Sample details, library names, and corresponding accessions for transcriptomes from the N_{short} experiment.

Files are available at the NCBI Sequence Read Archive (SRA) under the identifier PRJNA311568. Replicate experiments are indicated (A or B). Sample ID corresponds to label in Supplementary Dataset 1.

Rep.	Time point	N Source	Sample ID	Library	Accession
A	Pretreatment 1 (exp)	NH4+	Pre_1_A	NH4 (NH4A-PE-IL1-1)	SRX1922057
A	Pretreatment 2 (2 hr)	N-	Pre_2_A	N-free (NFREEA-PE-IL2-1)	SRX1922055
A	Pretreatment 3 (90 min)	NO3-	Pre_3_A	NO3 spike (NO3SPIKEA-PE-IL3-1)	SRX1922059
A	15 min	NO3-	NO3-_4_A	T15 NO3 (T15NO3A-PE-IL5-1)	SRX1922074
A	15 min	NO2-	NO2-_4_A	T15 NO2 (T15NO2A-PE-IL6-1)	SRX1922065
A	15 min	NH4+	NH4+_4_A	T15 NH4 (T15NH4A-PE-IL7-1)	SRX1922063
A	15 min	N-	N-_4_A	T15 N-free (T15NFREEA-PE-IL4-1)	SRX1922061
A	45 min	NO3-	NO3-_5_A	T45 NO3 (T45NO3A-PE-IL9-1)	SRX1922093
A	45 min	NO2-	NO2-_5_A	T45 NO2 (T45NO2A-PE-IL10-1)	SRX1922091
A	45 min	NH4+	NH4+_5_A	T45 NH4 (T45NH4A-PE-IL11-1)	SRX1922089
A	45 min	N-	N-_5_A	T45 N-free (T45NFREEA-PE-IL8-1)	SRX1922076
A	18 hr	NO3-	NO3-_6_A	T-end NO3 (TENDNO3A-PE-IL26-1)	SRX1922107
A	18 hr	NO2-	NO2-_6_A	T-end NO2 (TENDNO2A-PE-IL27-1)	SRX1922105
A	18 hr	NH4+	NH4+_6_A	T-end NH4 (TENDNH4A-PE-IL28-1)	SRX1922097
A	18 hr	N-	N-_6_A	T-end N-free (TENDNFREEA-PE-IL12-1)	SRX1922095
B	Pretreatment 1 (exp)	NH4+	Pre_1_B	NH4 (NH4B-PE-IL1-1)	SRX1922058
B	Pretreatment 2 (2 hr)	N-	Pre_2_B	N-free (NFREEB-PE-IL2-1)	SRX1922056
B	Pretreatment 3 (90)	NO3-	Pre_3_B	NO3 spike (NO3SPIKEB-PE-IL3-1)	SRX1922060
B	15 min	NO3-	NO3-_4_B	T15 NO3 (T15NO3B-PE-IL5-1)	SRX1922075
B	15 min	NO2-	NO2-_4_B	T15 NO2 (T15NO2B-PE-IL6-1)	SRX1922073
B	15 min	NH4+	NH4+_4_B	T15 NH4 (T15NH4B-PE-IL7-1)	SRX1922064
B	15 min	N-	N-_4_B	T15 N-free (T15NFREEB-PE-IL4-1)	SRX1922062
B	45 min	NO3-	NO3-_5_B	T45 NO3 (T45NO3B-PE-IL9-1)	SRX1922094
B	45 min	NO2-	NO2-_5_B	T45 NO2 (T45NO2B-PE-IL10-1)	SRX1922092
B	45 min	NH4+	NH4+_5_B	T45 NH4 (T45NH4B-PE-IL11-1)	SRX1922090
B	45 min	N-	N-_5_B	T45 N-free (T45NFREEB-PE-IL8-1)	SRX1922088
B	18 hr	NO3-	NO3-_6_B	T-end NO3 (TENDNO3B-PE-IL26-1)	SRX1922108
B	18 hr	NO2-	NO2-_6_B	T-end NO2 (TENDNO2B-PE-IL27-1)	SRX1922106
B	18 hr	NH4+	NH4+_6_B	T-end NH4 (TENDNH4B-PE-IL28-1)	SRX1922098
B	18 hr	N-	N-_6_B	T-end N-free (TENDNFREEB-PE-IL12-1)	SRX1922096

Supplementary Table 4: Sample details and corresponding accessions for proteome from the N_{short} experiment.

Files are available at the Proteomics IDEntifications (PRIDE) database under the identifier PXD015061. Samples are from N_{short} biological replicate experiment B. Pre-treatment samples: N-Free +2h B and NO3 SPIKE B. Nitrogen treatments correspond to N-free B, NO2 B, NO3 B, and NH4 B for nitrogen free, nitrite, nitrate, and ammonium respectively. T-15, T-45, and T-end correspond to 15 min, 45. Min, and 18 hr sampling times respectively.

**Dataset**	**Accession**	**113 ion**	**114 ion**	**115 ion**	**116 ion**	**117 ion**	**118 ion**	**119 ion**	**121 ion**
Andy0016_1_11Jul13_Pippin_12-12-39	112779	16, N-Free +2h B	18, NO3 SPIKE B	20, T-15 N-free B	22, T-15 NO3 B	28, T-45 N-Free B	30, T-45 NO3 B	36, T-end N-free B	38, T-end NO3 B
Andy0016_2_11Jul13_Pippin_12-12-39	112778	16, N-Free +2h B	18, NO3 SPIKE B	20, T-15 N-free B	22, T-15 NO3 B	28, T-45 N-Free B	30, T-45 NO3 B	36, T-end N-free B	38, T-end NO3 B
Andy0016_3_11Jul13_Pippin_12-12-39	112777	16, N-Free +2h B	18, NO3 SPIKE B	20, T-15 N-free B	22, T-15 NO3 B	28, T-45 N-Free B	30, T-45 NO3 B	36, T-end N-free B	38, T-end NO3 B
Andy0016_4_11Jul13_Pippin_12-12-39	112788	16, N-Free +2h B	18, NO3 SPIKE B	20, T-15 N-free B	22, T-15 NO3 B	28, T-45 N-Free B	30, T-45 NO3 B	36, T-end N-free B	38, T-end NO3 B
Andy0016_5_11Jul13_Pippin_12-12-39	112776	16, N-Free +2h B	18, NO3 SPIKE B	20, T-15 N-free B	22, T-15 NO3 B	28, T-45 N-Free B	30, T-45 NO3 B	36, T-end N-free B	38, T-end NO3 B
Andy0016_6_11Jul13_Pippin_12-12-39	112787	16, N-Free +2h B	18, NO3 SPIKE B	20, T-15 N-free B	22, T-15 NO3 B	28, T-45 N-Free B	30, T-45 NO3 B	36, T-end N-free B	38, T-end NO3 B
Andy0016_7_11Jul13_Pippin_12-12-39	112775	16, N-Free +2h B	18, NO3 SPIKE B	20, T-15 N-free B	22, T-15 NO3 B	28, T-45 N-Free B	30, T-45 NO3 B	36, T-end N-free B	38, T-end NO3 B
Andy0016_8_11Jul13_Pippin_12-12-39	112786	16, N-Free +2h B	18, NO3 SPIKE B	20, T-15 N-free B	22, T-15 NO3 B	28, T-45 N-Free B	30, T-45 NO3 B	36, T-end N-free B	38, T-end NO3 B
Andy0016_9_11Jul13_Pippin_12-12-39	112774	16, N-Free +2h B	18, NO3 SPIKE B	20, T-15 N-free B	22, T-15 NO3 B	28, T-45 N-Free B	30, T-45 NO3 B	36, T-end N-free B	38, T-end NO3 B
Andy0016_10_11Jul13_Pippin_12-12-39	112785	16, N-Free +2h B	18, NO3 SPIKE B	20, T-15 N-free B	22, T-15 NO3 B	28, T-45 N-Free B	30, T-45 NO3 B	36, T-end N-free B	38, T-end NO3 B
Andy0016_11_11Jul13_Pippin_12-12-39	112773	16, N-Free +2h B	18, NO3 SPIKE B	20, T-15 N-free B	22, T-15 NO3 B	28, T-45 N-Free B	30, T-45 NO3 B	36, T-end N-free B	38, T-end NO3 B
Andy0016_12_11Jul13_Pippin_12-12-39	112784	16, N-Free +2h B	18, NO3 SPIKE B	20, T-15 N-free B	22, T-15 NO3 B	28, T-45 N-Free B	30, T-45 NO3 B	36, T-end N-free B	38, T-end NO3 B
Andy0017_1_8Jul13_Pippin_12-12-39	112772	16, N-Free +2h B	18, NO3 SPIKE B	24, T-15 NO2 B	26, T-15 NH4 B	32, T-45 NO2 B	34, T-45 NH4 B	40, T-end NO2 B	42, T-end NH4 B
Andy0017_2_8Jul13_Pippin_12-12-39	112783	16, N-Free +2h B	18, NO3 SPIKE B	24, T-15 NO2 B	26, T-15 NH4 B	32, T-45 NO2 B	34, T-45 NH4 B	40, T-end NO2 B	42, T-end NH4 B
Andy0017_3_8Jul13_Pippin_12-12-39	112789	16, N-Free +2h B	18, NO3 SPIKE B	24, T-15 NO2 B	26, T-15 NH4 B	32, T-45 NO2 B	34, T-45 NH4 B	40, T-end NO2 B	42, T-end NH4 B
Andy0017_4_Rerun_11Jul13_Pippin_12-12-39	112794	16, N-Free +2h B	18, NO3 SPIKE B	24, T-15 NO2 B	26, T-15 NH4 B	32, T-45 NO2 B	34, T-45 NH4 B	40, T-end NO2 B	42, T-end NH4 B
Andy0017_5_8Jul13_Pippin_12-12-39	112771	16, N-Free +2h B	18, NO3 SPIKE B	24, T-15 NO2 B	26, T-15 NH4 B	32, T-45 NO2 B	34, T-45 NH4 B	40, T-end NO2 B	42, T-end NH4 B
Andy0017_6_8Jul13_Pippin_12-12-39	112782	16, N-Free +2h B	18, NO3 SPIKE B	24, T-15 NO2 B	26, T-15 NH4 B	32, T-45 NO2 B	34, T-45 NH4 B	40, T-end NO2 B	42, T-end NH4 B
Andy0017_7_8Jul13_Pippin_12-12-39	112793	16, N-Free +2h B	18, NO3 SPIKE B	24, T-15 NO2 B	26, T-15 NH4 B	32, T-45 NO2 B	34, T-45 NH4 B	40, T-end NO2 B	42, T-end NH4 B
Andy0017_8_8Jul13_Pippin_12-12-39	112781	16, N-Free +2h B	18, NO3 SPIKE B	24, T-15 NO2 B	26, T-15 NH4 B	32, T-45 NO2 B	34, T-45 NH4 B	40, T-end NO2 B	42, T-end NH4 B
Andy0017_9_8Jul13_Pippin_12-12-39	112792	16, N-Free +2h B	18, NO3 SPIKE B	24, T-15 NO2 B	26, T-15 NH4 B	32, T-45 NO2 B	34, T-45 NH4 B	40, T-end NO2 B	42, T-end NH4 B
Andy0017_10_8Jul13_Pippin_12-12-39	112780	16, N-Free +2h B	18, NO3 SPIKE B	24, T-15 NO2 B	26, T-15 NH4 B	32, T-45 NO2 B	34, T-45 NH4 B	40, T-end NO2 B	42, T-end NH4 B
Andy0017_11_8Jul13_Pippin_12-12-39	112991	16, N-Free +2h B	18, NO3 SPIKE B	24, T-15 NO2 B	26, T-15 NH4 B	32, T-45 NO2 B	34, T-45 NH4 B	40, T-end NO2 B	42, T-end NH4 B
Andy0017_12_8Jul13_Pippin_12-12-39	112790	16, N-Free +2h B	18, NO3 SPIKE B	24, T-15 NO2 B	26, T-15 NH4 B	32, T-45 NO2 B	34, T-45 NH4 B	40, T-end NO2 B	42, T-end NH4 B
