# Supplementary material for: Evolution and regulation of nitrogen flux through compartmentalized metabolic networks in a marine diatom
Source: Nat Commun. 2019 Oct 7;10:4552. doi: 10.1038/s41467-019-12407-y (PMC6779911; doi:10.1038/s41467-019-12407-y)
Supplement: Supplementary file 2 — Description of Additional Supplementary Files [file 41467_2019_12407_MOESM2_ESM.docx]

**Description of Supplementary Files**

**File Name: Supplementary Data 1**

**Description:** Nshort transcriptome. Phaeodactylum tricornutum (Phatr3) accessions (column A) coordinates (B-F) and annotations (G-AI). Subcellular localization targeting predictions based on HECTAR Gschloessl et al. 2008 (AJ - AO). Highly nitrate sensitive (HNS), nitrate sensitive (NS), nitratespecific response type (NO3 - RT), response type (RT), and proteome cluster (PC) identification (AP-AT). Calculations used for HNS determination, AR = Ammonia Repression, NIRA = Nitrate induction relative to ammonia, NIRN = Nitrate induction relative to nitrate (AU - AY). RPKM data (averages and per replicate, AZ - CS). EdgeR calculated differential expression data (CT - EO). Additional description of sample IDs and corresponding libraries can be found in Supplementary Table 3.

**File Name: Supplementary Data 2**

**Description:** TOMTOM Output.

**File Name: Supplementary Data 3**

**Description:** Nitrogen assimilation and metabolism gene catalog in Phaeodactylum tricornutum. Gene names and accessions are indicated, along with assigned subcellular localization based on summary of targeting predictions. Corresponding reaction in the Genome-scale reconstruction indicated in columns H-I. Notes on manual curation of gene models can be found in column Q.

**File Name: Supplementary Data 4**

**Description:** Modeled fluxes on nitrate (NO3 -) and urea. (after Levering et al. 2016, Broddrick

et al. 2018).
