# Supplementary material for: Evolution and regulation of nitrogen flux through compartmentalized metabolic networks in a marine diatom
Source: Nat Commun. 2019 Oct 7;10:4552. doi: 10.1038/s41467-019-12407-y (PMC6779911; doi:10.1038/s41467-019-12407-y)
Supplement: Supplementary file 7 — Reporting Summary [file 41467_2019_12407_MOESM7_ESM.pdf]

nature research

Corresponding author(s): Andrew E. Allen

Last updated by author(s): Aug 21, 2019

Reporting Summary

Nature Research wishes to improve the reproducibility of the work that we publish. This form provides structure for consistency and transparency in reporting. For further information on Nature Research policies, see Authors & Referees and the Editorial Policy Checklist.

Statistics

For all statistical analyses, confirm that the following items are present in the figure legend, table legend, main text, or Methods section.

n/a	Confirmed	
☐	☒	The exact sample size (*n*) for each experimental group/condition, given as a discrete number and unit of measurement
☐	☒	A statement on whether measurements were taken from distinct samples or whether the same sample was measured repeatedly
☐	☒	The statistical test(s) used AND whether they are one- or two-sided<br>*Only common tests should be described solely by name; describe more complex techniques in the Methods section.*
☒	☐	A description of all covariates tested
☒	☐	A description of any assumptions or corrections, such as tests of normality and adjustment for multiple comparisons
☐	☒	A full description of the statistical parameters including central tendency (e.g. means) or other basic estimates (e.g. regression coefficient) AND variation (e.g. standard deviation) or associated estimates of uncertainty (e.g. confidence intervals)
☐	☒	For null hypothesis testing, the test statistic (e.g. *F*, *t*, *r*) with confidence intervals, effect sizes, degrees of freedom and *P* value noted<br>*Give P values as exact values whenever suitable.*
☒	☐	For Bayesian analysis, information on the choice of priors and Markov chain Monte Carlo settings
☐	☒	For hierarchical and complex designs, identification of the appropriate level for tests and full reporting of outcomes
☒	☐	Estimates of effect sizes (e.g. Cohen's *d*, Pearson's *r*), indicating how they were calculated

Our web collection on statistics for biologists contains articles on many of the points above.

Software and code

Policy information about availability of computer code

Data collection	MASIC software v2.8.6303 was used to collect proteomics data and is publicly available.
Data analysis	Inferno v1.1 was used to analyze proteomics data and is publicly available. RNA-seq analysis was conducted with TopHat v.2.0.14 and EdgeR v 3.10.5.

For manuscripts utilizing custom algorithms or software that are central to the research but not yet described in published literature, software must be made available to editors/reviewers. We strongly encourage code deposition in a community repository (e.g. GitHub). See the Nature Research guidelines for submitting code & software for further information.

Data

Policy information about availability of data

All manuscripts must include a data availability statement. This statement should provide the following information, where applicable:

- Accession codes, unique identifiers, or web links for publicly available datasets
- A list of figures that have associated raw data
- A description of any restrictions on data availability

Nucleotide sequence data generated during this study are available are available at the NCBI Sequence Read Archive (SRA) under the identifier PRJNA311568. Mass spectrometry proteomics data have been deposited to the ProteomeXchange Consortium via the PRIDE partner repository52 with the dataset identifier PXD015061 and 10.6019/PXD015061[https://www.ebi.ac.uk/pride/archive/projects/PXD015061
]. Phylogenetic trees are publicly available via Figshare [https://figshare.com/s/600f55d61da0b39d2032]. Data underlying Figs 1a, 1b, 2c, 1d, 3b, 3c, 5, 7a, 7b, 8b, Supp Figs 1a, 1b, 2, 5a, 5b, 5c, 5d, and 6 are provided as Source Data files. All other data are available from the corresponding author upon request.

Field-specific reporting

Please select the one below that is the best fit for your research. If you are not sure, read the appropriate sections before making your selection.

☒ Life sciences ☐ Behavioural & social sciences ☐ Ecological, evolutionary & environmental sciences

For a reference copy of the document with all sections, see nature.com/documents/nr-reporting-summary-flat.pdf

Life sciences study design

All studies must disclose on these points even when the disclosure is negative.

Sample size	No statistical methods were used to predetermine sample size. The sample sizes were determined, in part, based on the availability of resources including time, cost, and personnel. All experiments were conducted in biological duplicate. Samples from the four treatment factor time course experiment were analyzed for transcriptomics (duplicate), proteomics (single replicate), and metabolomics (single replicate). Samples from the two factor time course experiment were analyzed for metabolomics/fluxomics (duplicate). This level of replication was sufficient to detect significant differential expression (fdr below a 0.05 threshold).
Data exclusions	No data were excluded from the analyses.
Replication	*Describe the measures taken to verify the reproducibility of the experimental findings. If all attempts at replication were successful, confirm this OR if there are any findings that were not replicated or cannot be reproduced, note this and describe why.*
Randomization	All n numbers represent biological replicates, and the experiments were not randomized or blinded. There was no biased use of particular tubes or reagents that was correlated with experimental conditions. Care was taken to repeat experimental measurements in an unbiased way so as to mitigate the effect of cofactors such as sampling tubes, assay instrumentation, and reagents.
Blinding	Not applicable. Group allocation was not a feature for experiments and associated data.

Reporting for specific materials, systems and methods

We require information from authors about some types of materials, experimental systems and methods used in many studies. Here, indicate whether each material, system or method listed is relevant to your study. If you are not sure if a list item applies to your research, read the appropriate section before selecting a response.

Materials & experimental systems

n/a	Involved in the study	
☐	☒	Antibodies
☐	☒	Eukaryotic cell lines
☒	☐	Palaeontology
☒	☐	Animals and other organisms
☒	☐	Human research participants
☒	☐	Clinical data

Methods

n/a	Involved in the study	
☒	☐	ChIP-seq
☒	☐	Flow cytometry
☒	☐	MRI-based neuroimaging

Antibodies

Antibodies used	AbCam Anti-GRF antibody ab1218
Validation	From Manufacturer: Assayed by ELISA for direct binding of antigen recognizes wild type and recombinant GFP. Well suited to titrate GFP in solution using either form of the antibody as the capture or detection antibodies. Shows no reactivity against red fluorescence proteins (RFP). This antibody is known to cross react with the wild type (wt), recombinant (rGFP) and enhanced (eGFP) forms.

Eukaryotic cell lines

Policy information about cell lines

Cell line source(s)	Phaeodactylum tricornutum strain CCMP 632 was obtained from the National Center for Marine Algae and Microbiota. Transgenic lines of this strain expressing eYFP fusion proteins were generated (details in methods).
Authentication	The wild-type strain was not authenticated. Transgenic lines were authenticated for the presence of transgene by PCR.
Mycoplasma contamination	Cell lines were not tested for Mycoplasma contamination.

Commonly misidentified lines<br>(See ICLAC register)	No commonly misidentified cell lines were used.
